# RNA viruses promote activation of the NLRP3 inflammasome through cytopathogenic effect-induced potassium efflux

**DOI:** 10.1038/s41419-019-1579-0

**Published:** 2019-04-25

**Authors:** Leandro Silva da Costa, Ahmed Outlioua, Adrienne Anginot, Khadija Akarid, Damien Arnoult

**Affiliations:** 1 INSERM, UMR_S 1197, Hôpital Paul Brousse, Villejuif, France; 2 Université Paris-Saclay, Paris, France; 3 Molecular Genetics and Immunophysiopathology Research Team, Health and Environment Laboratory, Aïn Chock Faculty of Sciences, Hassan II University of Casablanca, Casablanca, Morocco

**Keywords:** Infection, NOD-like receptors

## Abstract

Early detection of viruses by the innate immune system is crucial for host defense. The NLRP3 inflammasome, through activation of caspase-1, promotes the maturation of IL-1β and IL-18, which are critical for antiviral immunity and inflammatory response. However, the mechanism by which viruses activate this inflammasome is still debated. Here, we report that the replication of cytopathogenic RNA viruses such as vesicular stomatitis virus (VSV) or encephalomyocarditis virus (EMCV) induced a lytic cell death leading to potassium efflux, the common trigger of NLRP3 inflammasome activation. This lytic cell death was not prevented by a chemical or genetic inhibition of apoptosis, pyroptosis, or necroptosis but required the viral replication. Hence, the viruses that stimulated type I IFNs production after their sensing did not activate NLRP3 inflammasome due to an inhibition of their replication. In contrast, NLRP3 inflammasome activation induced by RNA virus infection was stimulated in IFNAR-deficient or MAVS-deficient cells consequently to an increased viral replication and ensuing lytic cell death. Therefore, in a context of inefficient IFN response, viral replication-induced lytic cell death activates of the NLRP3 inflammasome to fight against infection.

## Introduction

Besides directing adaptive immune responses, the pleiotropic cytokines interleukin-1β (IL-1β) and IL-18 play an essential role in inflammatory responses^1^. The production of both cytokines is a two-step process, as it is regulated at the transcriptional as well as the posttranslational level. Hence, a pathogen or a danger molecule that causes the secretion of IL-1β and IL-18 has to provide both signals. Indeed, while pro-IL-18 is constitutively expressed, pro-IL-1β expression is induced by NF-κB, for instance after toll-like receptor activation. Next pro-IL-1β and pro-IL-18 are matured and processed by caspase-1. Activation of this inflammatory caspase is regulated by a complex of proteins called the inflammasome. The cytosolic receptors of the NACHT and LRR-containing gene (NLR) family are components of most inflammasomes, and these receptors are indirectly coupled to caspase-1 through the adaptor apoptosis-associated speck (ASC-like protein containing a CARD)^2^. Detection of an agonist by an NLR promotes its oligomerization and recruits caspase-1 leading to its activation after a proteolytic processing. Once activated, caspase-1 also cleaves the N-terminal of gasdermin D to promote pyroptosis, a lytic form of cell death^2^. Inflammasomes are now recognized for their crucial roles in host defense against pathogens^3^, but abnormal inflammasome activations are also associated with several pathologies as the development of some cancers—neurodegenerative, autoimmune, and metabolic diseases.

Several inflammasomes have been identified^2^. Unlike the other inflammasomes that sense/detect specific agonists, the NLRP3 inflammasome is activated by different signals as crystals (Alum, CPPD, MSU, etc.), extracellular ATP, nucleic acids, bacteria, fungi, viruses, etc.^2^. While the mechanisms of NLRP3 inflammasome is still debated^4^, the efflux of intracellular K^+^ appears as a necessary and sufficient upstream signaling event in NLRP3 activation^2,5^.

Since they are critical components of the innate immunity, the inflammasomes have been reported to play an important role in host defense by sensing viral infection and promoting responses from the innate immune system^6^. After infection with a DNA virus as vaccinia virus or murine cytomegalovirus, the AIM2 inflammasome is activated in response to the cytosolic double-stranded DNA^7,8^. The NLRP3 inflammasome senses infection of some DNA viruses and RNA viruses like adenovirus, vesicular stomatitis virus (VSV), or influenza virus^9–11^. Nevertheless, the mechanism by which NLRP3 perceives viral infection remains debated and unclear as ion flux induced by the influenza virus-encoded M2 channel, detection of viral RNA, or involvement of a RIPK1–RIPK3–Drp1 signaling pathway have been proposed^6,12,13^.

In this study, we report that the cytopathogenic RNA viruses like VSV or encephalomyocarditis virus (EMCV) promote activation of the NLRP3 inflammasome as a consequence of a K^+^ efflux induced by the lytic cell death triggered by their replication.

## Results

### Cytopathogenic RNA viruses promote NLRP3 inflammasome activation

**Fig. 1:**
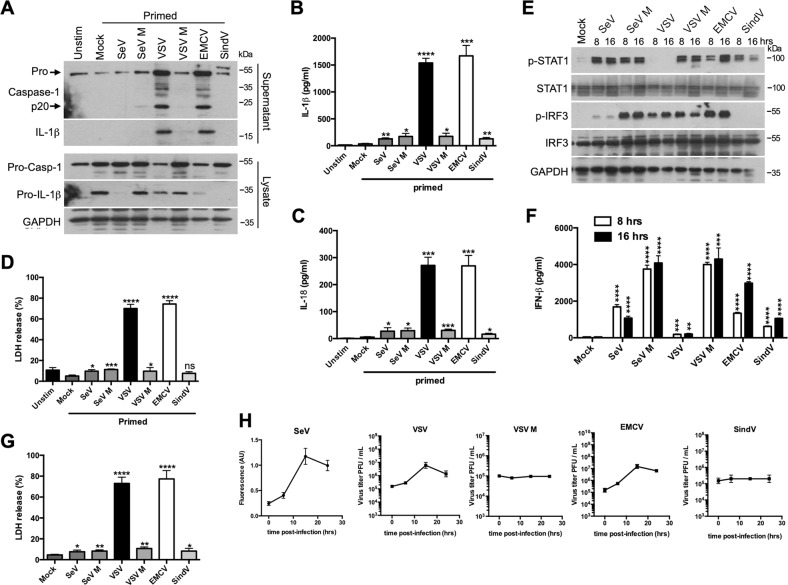
Replicating and cytopathogenic RNA viruses trigger inflammasome activation. **a** Primed (LPS, 100 ng/ml for 3 h) BMDMs were infected with different RNA viruses for 15 h. Then, cell supernatants and cell lysates were analyzed by WB for the indicated proteins. **b, c** Primed BMDMs were infected with different RNA viruses. After15 h, IL-1β (**b**) or IL-18 release (**c**) in the cell supernatant was assessed by ELISA. The data shown are means ± SD from three independent experiments (analysis of variance and comparison with mock BMDMs in Student’s *t-*test). ****P < 0.0001, ***P < 0.001, **0.001 < *P* < 0.01, *0.01 < *P* < 0.05. **d** Primed BMDMs were infected with different RNA viruses. After 15 h, cell death was assessed using the LDH release assay. The data shown are means ± SD from three independent experiments (analysis of variance and comparison with mock BMDMs in Student’s *t-*test). ****P < 0.0001, ***P < 0.001, *0.01 < *P* < 0.05. ns, not significant. **e** BMDMs were infected with different RNA viruses for 8 or 16 h. Then, cell lysates were analyzed by WB for the indicated proteins. **f** BMDMs were infected with different RNA viruses for 8 or 16 h. Then, IFNβ production was assessed by ELISA. The data shown are means ± SD from three independent experiments (analysis of variance and comparison with mock BMDMs in Student’s *t-*test). ****P < 0.0001, ***P < 0.001, **0.001 < *P* < 0.01. **g** BMDMs were infected with different RNA viruses. After 15 h, cell death was assessed using the LDH release assay. The data shown are means ± SD from three independent experiments (analysis of variance and comparison with mock BMDMs in Student’s *t-*test). ****P < 0.0001, **0.001 < *P* < 0.01, *0.01 < *P* < 0.05. **h** Analysis of the viral replication in BMDMs

Although previous investigations have demonstrated that some RNA viruses trigger NLRP3 inflammasome activation^14,15^, few works have compared in the same study the inflammasome activation through different RNA viruses. For this purpose, the paramyxoviridae Sendai virus (SeV), Sendai virus H4 strain (SeV M) composed mostly of small copyback defective interfering genomes overproducing pppRNAs, and underproducing viral V and C protein^16^, the rhabdoviridae VSV, VSV with a mutation in matrix (M) protein (VSV M)^17^, the picornaviridae EMCV, and the alphavirus Sindbis virus (SindV) were used. LPS-primed bone marrow-derived macrophages (BMDMs) were infected with the different viruses, next the presence of the p20 of the active caspase-1 and IL-1β was assessed by WB in the supernatant of the infected cells (Fig. 1a). p20 and mature IL-1β were only detected in the supernatant of VSV-infected or EMCV-infected BMDMs (Fig. 1a). In primed BMDMs infected with SeV or SinV, pro-IL-1β was less expressed (Fig. 1a), but it does not seem to be due to an inhibition of the NF-κB signaling or to depend on IFN-β (Fig. S1). The significant production of pro-inflammatory cytokine IL-1β after VSV or EMCV infection was further confirmed by enzyme-linked immunosorbent assays (ELISA) (Fig. 1b) as well as the secretion of IL-18 (Fig. 1c). Caspase-1 activation not only promotes the release of pro-inflammatory cytokines but also triggers a form of cell death called pyroptosis through the cleavage of gasdermin D^2,18^. So, the release of both cytokines was associated with cell death in VSV-infected or EMCV-infected cells (Fig. 1d). Interestingly, the capability of RNA viruses to trigger inflammasome activation seems to be independent of their sensing by the innate immunity machinery. Indeed VSV, unlike EMCV, did not promote IFN-β production in infected BMDMs (Fig. 1e, f), while both viruses were excellent inflammasome inducers (Fig. 1a–c). BMDMs without priming were infected with the different RNA viruses and cell death was detected only after infection with VSV or EMCV (Fig. 1g). Given that both viruses replicated very well in BMDMs (Fig. 1h), our observations suggest therefore that only the replicating viruses with a cytopathogenic effect like VSV or EMCV are efficient in inducing inflammasome activation (Fig. 1g, h). VSV or EMCV infection was not capable per se to trigger pro-IL-1β expression and prime NLRP3 inflammasome in vitro (Fig. S2), in agreement with a previous study^11^.

**Fig. 2:**
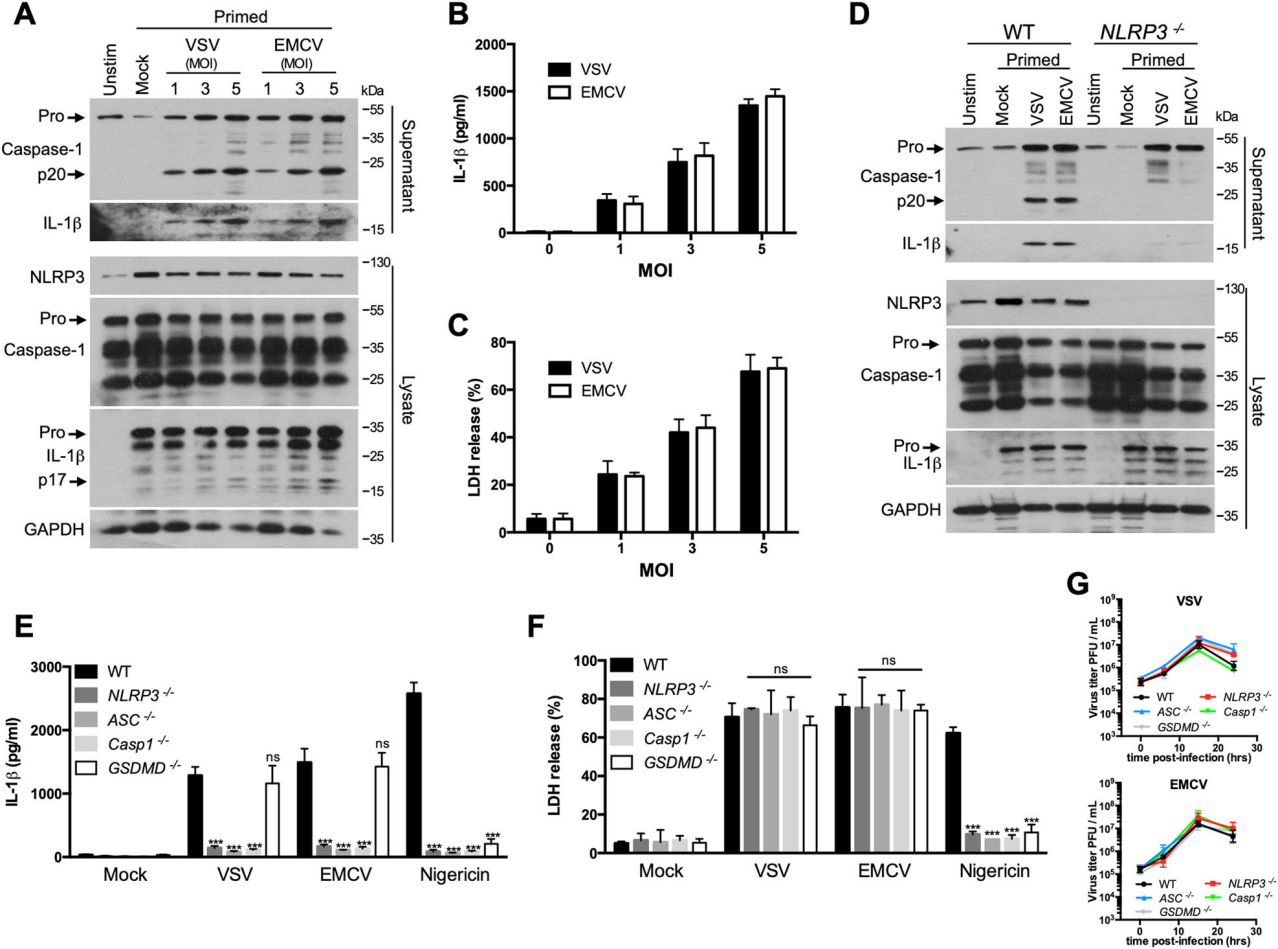
VSV and EMCV promote NLRP3 inflammasome activation and inflammasome-independent cell death. **a** Primed BMDMs were infected with increased MOI of VSV or EMCV for 15 h. Then, cell supernatants and cell lysates were analyzed by WB for the indicated proteins. **b** Primed BMDMs were infected with increased MOI of VSV or EMCV. After 15 h, IL-1β release in the cell supernatant was assessed by ELISA. **c** Primed BMDMs were infected with increased MOI of VSV or EMCV. After 15 h, cell death was assessed using the LDH release assay. **d** Primed WT or NLRP3^−/−^ BMDMs were infected with VSV or EMCV for 15 h. Then, cell supernatants and cell lysates were analyzed by WB for the indicated proteins. **e** Primed WT, NLRP3^−/−^, ASC^−/−^, Casp1^−/−^, or GSDMD^-/−^ BMDMs were infected with VSV or EMCV. After 15 h, IL-1β release in the cell supernatant was assessed by ELISA. The data shown are means ± SD from three independent experiments. ***P < 0.001 versus WT BMDMs (Student’s *t*-test). ns, not significant. **f** Primed WT, NLRP3^−/−^, ASC^−/−^, Casp1^−/−^, or GSDMD^−/−^ BMDMs were infected with VSV or EMCV. After 15 h, cell death was assessed using the LDH release assay. The data shown are means ± SD from three independent experiments. ****P < 0.0001 versus WT BMDMs (Student’s *t*-test). ns, not significant. **g** Analysis of the viral replication in the different BMDMs

Infection of primed BMDMs with increased MOI of VSV or EMCV led to increased caspase-1 maturation, IL-1β production, and cell death (Fig. 2a–c). As previously reported^11^, VSV or EMCV infection triggered NLRP3 inflammasome activation because caspase-1 activation was not observed and IL-1β release was severely blunted when NLRP3^−/−^ BMDMs were infected with the viruses (Fig. 2d, e). As expected, the genetic deletion of the adaptor ASC or of caspase-1 strongly inhibited IL-1β production (Fig. 2e). Following treatment with a toxin like nigericin, a classical NLRP3 activator, NLRP3 inflammasome formation leads to the recruitment and caspase-1 activation through the adaptor ASC, then active caspase-1 processes pro-IL-1β to its mature forms (Fig. S3) and cleaves gasdermin D (GSDMD)^2,18^. The N-terminal fragment of gasdermin D drives pyroptosis and allows the release of mature IL-1β from the cell. Hence, both IL-1β release and pyroptosis were strongly prevented in GSDMD^−/−^ BMDMs following nigericin treatment (Fig. 2e, f). Interestingly, IL-1β release was not impaired in GSDMD^−/−^ BMDMs infected with VSV or EMCV (Fig. 2e), suggesting that VSV or EMCV-mediated IL-1β release is independent of gasdermin D. Furthermore, the virus-induced cell death did not seem to be dependent on any components of the inflammasome, since cell death was not affected by the absence of NLRP3, ASC, caspase-1, or gasdermin D, unlike the treatment with nigericin (Fig. 2f). Finally, the observed effects were not due to altered viral replications as VSV or EMCV replication within the different BMDMs was normal (Fig. 2g).

VSV or EMCV infection induced the release of many proteins into the cell culture medium, and the release was unaffected by the absence of GSDMD (Fig. S4). The profile of release was quite similar when BMDMs were incubated in water to trigger necrosis (Fig. S4). These observations suggest that IL-1β and IL-18 release, like other cytosolic proteins, is simply due to plasma membrane rupture (a hallmark of necrosis) as a consequence of the lytic cell death induced by the replication of the cytopathogenic viruses.

### Cytopathogenic RNA viruses promotes potassium efflux to activate NLRP3 inflammasome

**Fig. 3:**
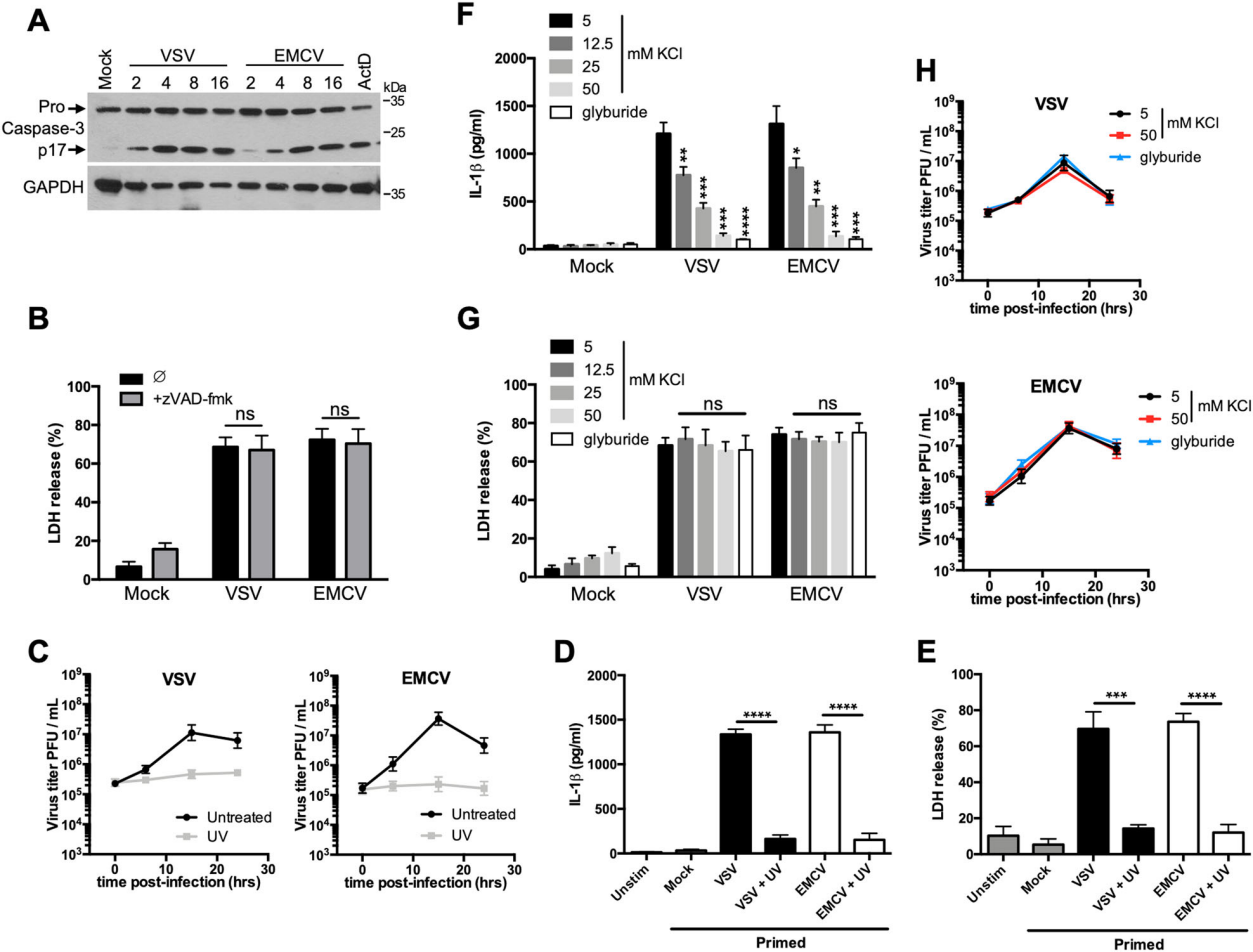
Replicating VSV and EMCV promote caspase-independent cell death and NLRP3 inflammasome activation through K^+^ efflux. **a** BMDMs were infected with VSV or EMCV. At different times after infection, cell lysates were analyzed by WB for the indicated proteins. Actinomycin D treatment (ActD) (20 μM for 8 h) was used as a positive control. **b** BMDMs were infected with VSV or EMCV in the presence or the absence of the pan caspase inhibitor zVAD-fmk (10 μM) for 15 h. Cell death was then assessed using the LDH release assay. The data shown are means ± SD from three independent experiments. ns, not significant. **c** Analysis of VSV or EMCV replication, either untreated or UV inactivated. **d** Primed BMDMs were infected with untreated or UV-inactivated VSV or EMCV for 15 h. Next, IL-1β release in the cell supernatant was assessed by ELISA. The data shown are means ± SD from three independent experiments. ****P < 0.001 versus untreated virus (Student’s *t*-test). **e** Primed BMDMs were infected with untreated or UV-inactivated VSV or EMCV. After 15 h, cell death was assessed using the LDH release assay. The data shown are means ± SD from three independent experiments. ****P *<* 0.0001, ***P < 0.001 versus untreated virus (Student’s *t*-test). **f** Primed BMDMs were infected with VSV or EMCV for 15 h in the presence of increased concentrations of KCl or glyburide (a proton pump inhibitor that prevents the K^+^ efflux, 25 μg/ml). IL-1β release in the cell supernatant was then assessed by ELISA. The data shown are means ± SD from three independent experiments. ****P *<* 0.0001, ***P < 0.001, **0.001 < *P* < 0.01, *0.01 < *P* < 0.05 versus 5 mM KCl (Student’s *t*-test). **g** Primed BMDMs were infected with VSV or EMCV for 15 h in the presence of increased concentrations of KCl or glyburide (25 μg/ml). Then, cell death was assessed using the LDH release assay. The data shown are means ± SD from three independent experiments. ns, not significant versus 5 mM KCl (Student’s *t*-test). **h** Analysis of the viral replication in the presence of KCl or glyburide

Elimination of infected cells via apoptosis is one of the most ancestral defense mechanism against viral infection^19^. VSV or EMCV infection promoted rapidly activation of the apoptotic effector caspase-3 (Fig. 3a). However, the use of a broad caspase inhibitor like zVAD-fmk did not prevent the virus-induced cell death (Fig. 3b). Finally, caspase inhibition did not affect ASC speck formation and ASC oligomerization (Fig. S5) after infection, indicating that the NLRP3 inflammasome formation and activation is independent of any caspase activity.

As shown in Figs. 1 and 2, infection with VSV or EMCV generates NLRP3 inflammasome activation and ensuing IL-1β release. However, when both viruses were UV-inactivated, preventing therefore their replication (Fig. 3c), IL-1β secretion from primed BMDMs was strongly reduced (Fig. 3d), indicating that the viral replication is required for VSV or EMCV to be “sensed” by the NLRP3 inflammasome. Moreover, UV-inactivated viruses barely induced cell death (Fig. 3e), suggesting again that cell death consequent to viral replication is required for inflammasome activation.

As we observed that the replication of VSV or EMCV within BMDMs promotes a form of necrosis (Fig. S4), we then hypothesized that this plasma membrane rupture allows an efflux of intracellular K^+^, a necessary and sufficient upstream signaling event in NLRP3 activation^2,5^. Confirming this, blocking K^+^ efflux by incubating cells in increased concentrations of KCl or with the proton pump inhibitor glyburide prevented IL-1β secretion after VSV or EMCV infection (Fig. 3f), without however preventing the cell death (Fig. 3g) or the viral replication (Fig. 3h). These observations indicate therefore that the viral replication promotes necrotic/lytic cell death and K^+^ efflux leading to NLRP3 inflammasome activation and IL-1β maturation.

### RLRs, MAVS, and DHX33 are not required for inflammasome activation by RNA viruses

**Fig. 4:**
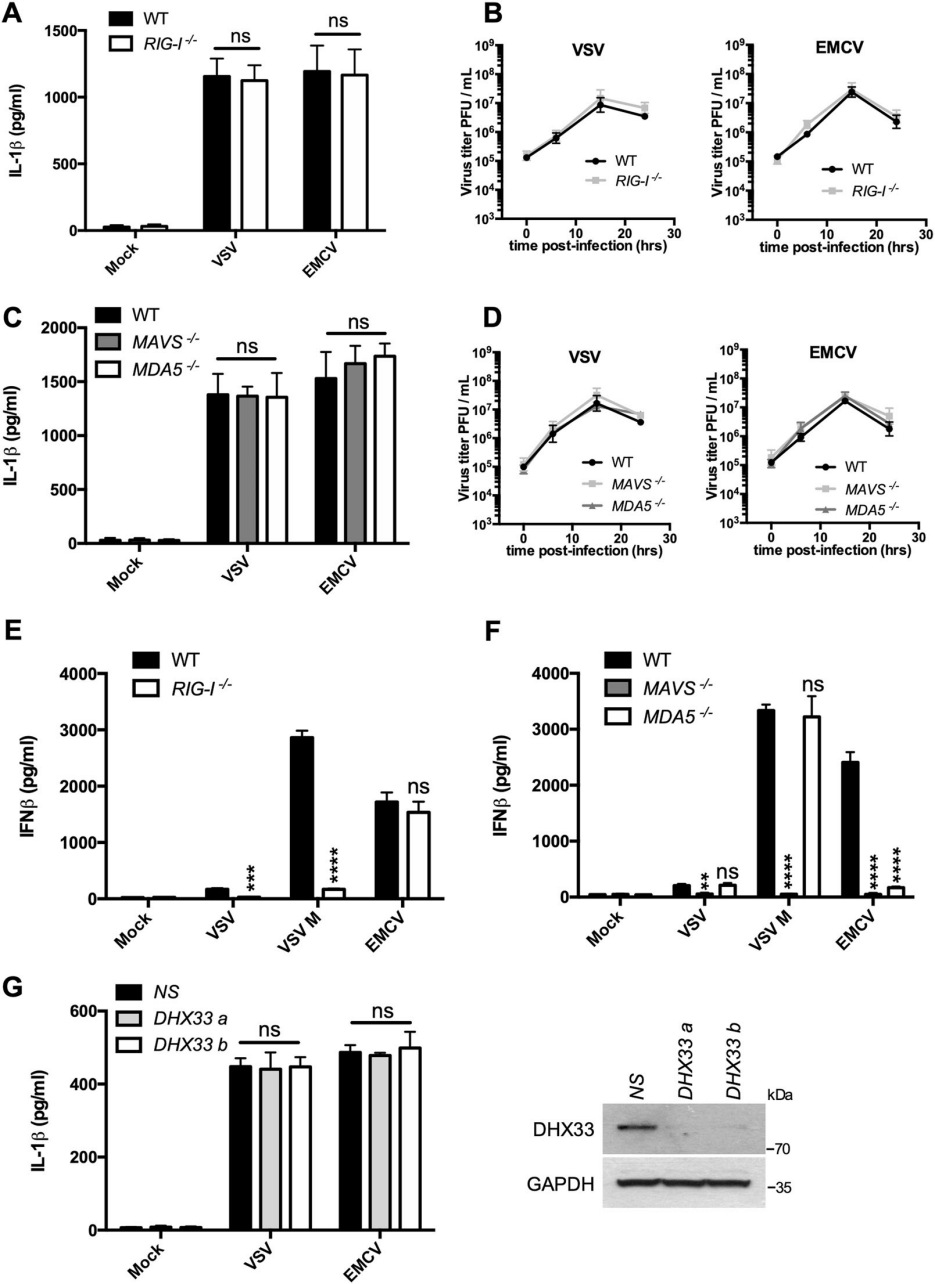
Virus-induced inflammasome activation is independent of RNA sensing by RIG-I, MDA-5, or DXH33. **a** Primed WT or RIG-I^−/−^ BMDMs were infected with VSV or EMCV. After 15 h, IL-1β release in the cell supernatant was assessed by ELISA. The data shown are means ± SD from three independent experiments. ns, not significant versus WT BMDMs (Student’s *t*-test). **b** Analysis of the viral replication in WT or RIG-I^−/−^ BMDMs. **c** Primed WT, MAVS^−/−^, or MDA5^−/−^ BMDMs were infected with VSV or EMCV. After 15 h, IL-1β release in the cell supernatant was assessed by ELISA. The data shown are means ± SD from three independent experiments. ns, not significant versus WT BMDMs (Student’s *t*-test). **d** Analysis of the viral replication in WT, MAVS^−/−^, or MDA5^−/−^ BMDMs. **e** WT or RIG-I^−/−^ BMDMs were infected with VSV, VSV M, or EMCV for 8 h. IFNβ release in the cell supernatant was next assessed by ELISA. The data shown are means ± SD from three independent experiments. ****P *<* 0.0001, ***P < 0.001, ns, not significant versus WT BMDMs (Student’s *t*-test). **f** WT, MAVS^−/−^, or MDA5^−/−^ BMDMs were infected with VSV, VSV M, or EMCV for 8 h. IFNβ release in the cell supernatant was assessed by ELISA. The data shown are means ± SD from three independent experiments. ****P *<* 0.0001, **0.001 < *P* < 0.01, ns, not significant versus WT BMDMs (Student’s *t*-test). **g** BMDMs were transfected with siRNAs raised against DHX33 or nonspecific siRNA (NS). After 3 days, BMDMs were infected with VSV or EMCV for 15 h. IL-1β release in the cell supernatant was then assessed by ELISA. The data shown are means ± SD from three independent experiments. ns, not significant versus NS siRNA-transfected BMDMs (Student’s *t*-test). The efficiency of the knockdown of DHX33 was confirmed by WB

RIG-I-like receptors (RLRs) play a central role in the innate immune response to RNA viral infection^20,21^. Whether RLR signaling played a role in inflammasome activation was then investigated. Neither RIG-I nor MDA5 was required for IL-1β secretion in response to VSV or EMCV and the absence of RLRs did not affect the replication (Fig. 4a–d). As controls, the production of IFNβ in response to VSV M required RIG-I (VSV being a really weak IFN-β inducer), while secretion of IFNβ in response to EMCV required MDA5 as previously reported^11,20,22^ (Fig. 4e, f). Our results indicate that RIG-I and MDA-5 do not seem to play a role in inflammasome activation in response to VSV or EMCV.

MAVS, the mitochondrial adaptor downstream of RLRs required for the signal transduction leading to the production of type I IFNs^23,24^ (Fig. 4f) has also been proposed to be required in NLRP3 inflammasome activation^25,26^. On the other hand, IL-1β release was not affected in MAVS^−/−^ BMDMs infected with VSV or EMCV (Fig. 4c). Likewise, the loss of MAVS did not reduce caspase-1 activation and IL-1β maturation in primed BMDMs after nigericin or ATP treatment (Fig. S6).

Finally, the DHX33 RNA helicase has been reported to sense cytosolic RNA and activate the NLRP3 inflammasome in human macrophages^27^, but IL-1β secretion in VSV-infected or EMCV-infected BMDMs was not affected after the knockdown of this helicase with siRNAs (Fig. 4g). The knockdown of DHX33 did not alter the viral replication (Fig. S7). In THP-1 cells, the knockdown of DHX33 did not impair IL-1β secretion after VSV or EMCV infection either, in agreement with another study^13^ (Fig. S8), but it did after Poly(I:C) transfection, as reported^27^.

### Necroptosis machinery and Drp1 are dispensable for inflammasome activation by RNA viruses

Besides apoptosis, necroptosis is another host defense strategy to prevent viral infection^28–30^. Hence, several viruses have been reported to induce necroptosis after infection^29,30^. Necroptosis is a form of a regulated cell death involving proteins containing a RHIM domain (RIPK1, TRIF, or ZPB1/DAI) allowing the recruitment of RIPK3 that ultimately activates the necroptosis executioner MLKL through phosphorylation^28^.

**Fig. 5:**
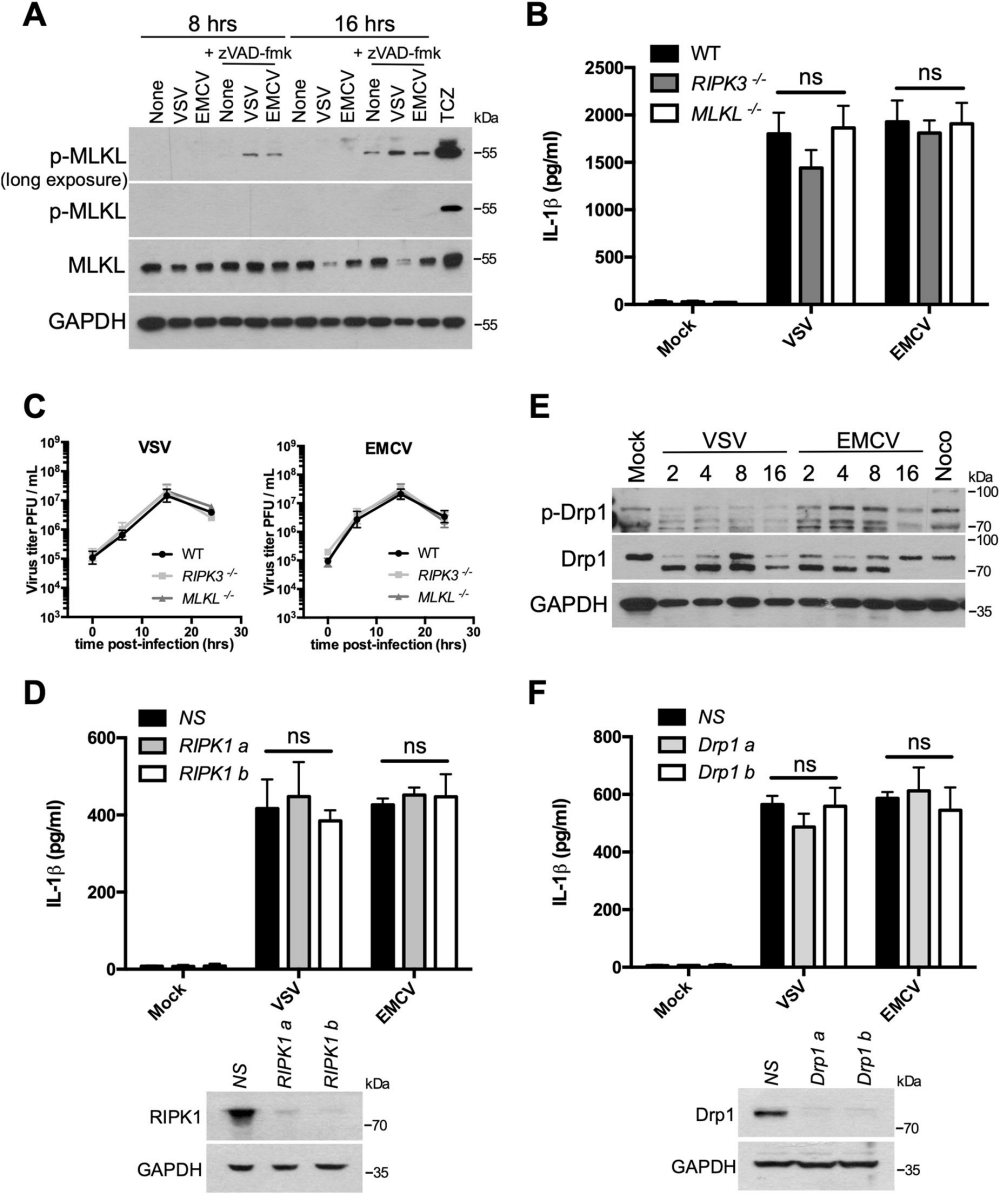
Virus-induced inflammasome activation is independent of necroptosis machinery and Drp1. **a** BMDMs were infected in the presence or the absence of zVAD-fmk (20 μM) with VSV or EMCV for 8 or 16 h. Then, cell lysates were analyzed by WB for the indicated proteins. As a positive control, BMDMs were treated with TNFα (50 ng/ml), cycloheximide (1 μg/ml), and zVAD-fmk (20 μM) (TCZ) for 2 h. **b** Primed WT, RIPK3^−/−^, or MLKL^−/−^ BMDMs were infected with VSV or EMCV. After 15 h, IL-1β release in the cell supernatant was assessed by ELISA. The data shown are means ± SD from three independent experiments. ns, not significant versus WT BMDMs (Student’s *t*-test). **c** Analysis of the viral replication in WT, RIPK3^−/−^, or MLKL^−/−^ BMDMs. **d** BMDMs were transfected with siRNAs raised against RIPK1 or nonspecific siRNA (NS). After 3 days, BMDMs were infected with VSV or EMCV for 15 h. IL-1β release in the cell supernatant was then assessed by ELISA. The data shown are means ± SD from three independent experiments. ns, not significant versus NS siRNA-transfected BMDMs (Student’s *t*-test). The efficiency of the knockdown of RIPK1 was confirmed by WB. **e** BMDMs were infected with VSV or EMCV for the indicated times. Cell lysates were analyzed by WB for the indicated proteins. As a positive control, BMDMs were treated with nocodazole (100 ng/ml) for 16 h. **f** BMDMs were transfected with siRNAs raised against Drp1 or nonspecific siRNA (NS). After 3 days, BMDMs were infected with VSV or EMCV for 15 h. IL-1β release in the cell supernatant was then assessed by ELISA. The data shown are means ± SD from three independent experiments. ns, not significant versus NS siRNA-transfected BMDMs (Student’s *t*-test). The efficiency of the knockdown of Drp1 was confirmed by WB

Given that our results suggested that the cell death induced by VSV or EMCV was independent of caspases, we have explored whether the viruses that promoted inflammasome activation, might trigger necroptosis as well. MLKL phosphorylation is a hallmark of necroptosis but VSV or EMCV infection did not cause its phosphorylation (Fig. 5a). Necroptosis is mainly activated under apoptosis-deficient conditions^28^, so only incubation with the broad caspase inhibitor zVAD-fmk yielded a weak MLKL phosphorylation (Fig. 5a). To rule out the possibility that necroptosis is the form of cell death promoting the K^+^ efflux involved in NLRP3 activation after viral infection, MLKL^−/−^ BMDMs were infected with VSV or EMCV but IL-1β release as well as replication were similar to the infected WT BMDMs (Fig. 5b, c). It has been proposed that the RNA viruses promote NLRP3 inflammasome activation through a RIPK1–RIPK3–Drp1 signaling pathway^13^. On the other hand, the loss of RIPK3 did not significantly affect IL-1β secretion and viral replication after VSV or EMCV infection (Fig. 5b, c), and ASC speck formation or ASC oligomerization was not significantly affected (Fig. S5). Furthermore, simultaneous inhibition of apoptosis and necroptosis did not alter ASC speck formation or ASC oligomerization (Fig. S5), indicating that NLRP3 activation in the context of cytopathogenic virus infection is independent of these forms of programmed cell death. RIPK1^−/−^ mice are not viable^31^ so that RIPK1 was knocked down in BMDMs using siRNA (Fig. 5d) and again, in agreement with a recent paper^32^, the knockdown of RIPK1 did not impact the inflammasome nor the viral replication following infection (Fig. 5d, S7), while it did prevent MLKL phosphorylation following necroptosis induction (Fig. S9A). RIPK1–RIPK3 following RNA virus infection were reported to induce phosphorylation/activation of Drp1^13^, an effector of the mitochondrial fission machinery^33^. While infection of BMDMs with EMCV led to an increased Drp1 phosphorylation (Fig. 5e), in contrast Drp1 phosphorylation was mitigated in VSV-infected cells (Fig. 5e), suggesting that Drp1 phosphorylation is not a hallmark for RNA virus-induced inflammasome activation. Moreover, the knockdown of Drp1 in BMDMs (Fig. 5f, S9B) did not affect IL-1β production after VSV or EMCV infection (Fig. 5f), as previously reported^32^. The knockdown of Drp1 did not affect the viral replication either (Fig. S7). Our observations suggest therefore that neither RIPK1, RIPK3, MLKL, nor Drp1 are required for inflammasome activation by RNA viruses.

### Viral replication and ensuing cell death are required for inflammasome activation

**Fig. 6:**
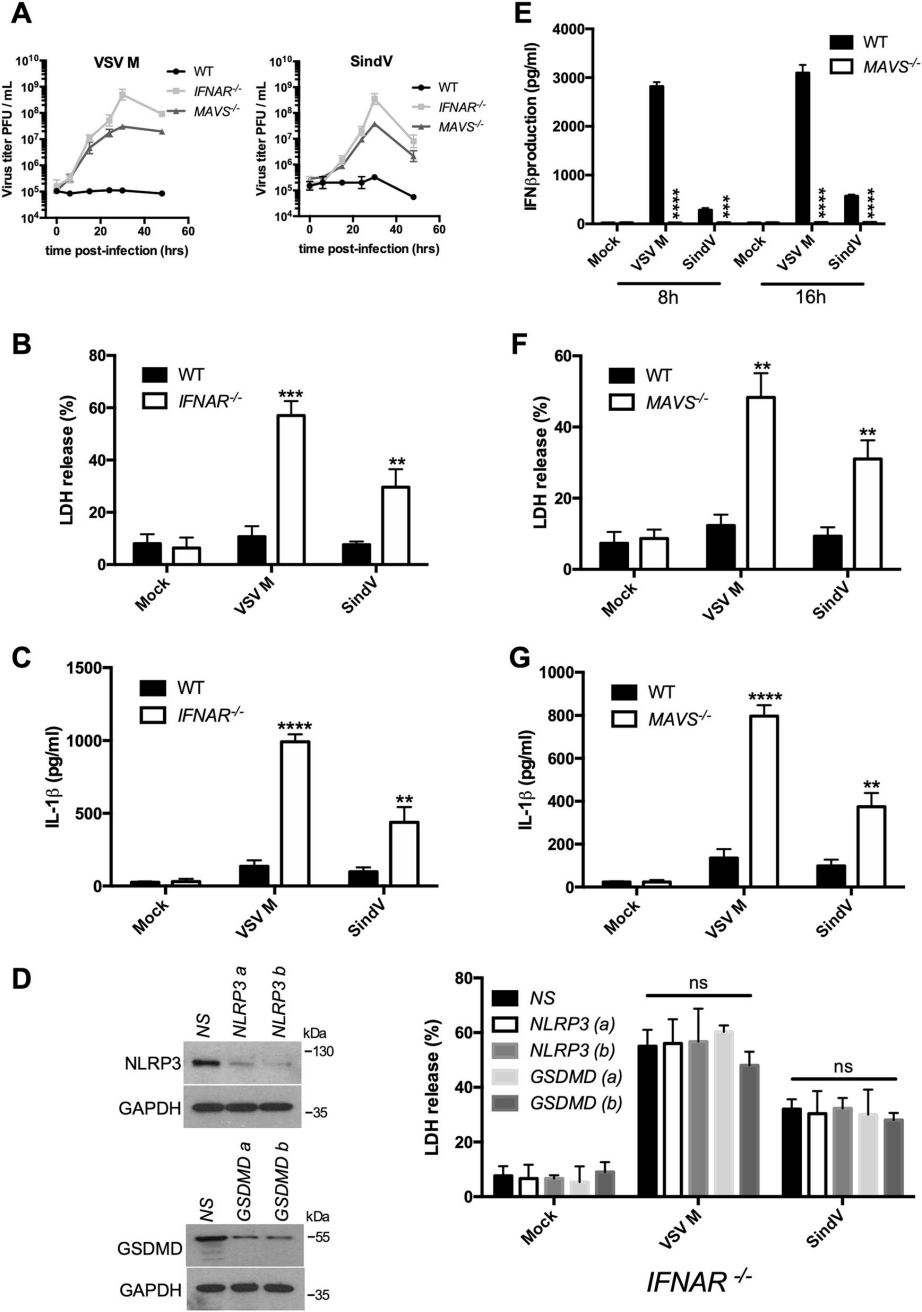
Viral replication is required for NLRP3 inflammasome activation. **a**VSV M or Sindbis virus (SindV) replication was assessed in WT, IFNAR^−/−^, or MAVS^−/−^ BMDMs. **b** WT or IFNAR^−/−^ BMDMs were infected with VSV M or SindV for 24 h, then cell death was assessed using the LDH release assay. The data shown are means ± SD from three independent experiments. ***P < 0.001, **0.001 < *P* < 0.01, versus WT BMDMs (Student’s *t*-test). **c** Primed WT or IFNAR^−/−^ BMDMs were infected with VSV M or SindV for 24 h. IL-1β release in the cell supernatant was then assessed by ELISA. The data shown are means ± SD from three independent experiments. ****P < 0.0001, **0.001 < *P* < 0.01, versus WT BMDMs (Student’s *t*-test). **d** IFNAR^−/−^ BMDMs were transfected with siRNAs raised against NLRP3 or GSDMD or nonspecific siRNA (NS). After 3 days, BMDMs were infected with VSV M or SindV for 24 h, then cell death was assessed using the LDH release assay. The data shown are means ± SD from three independent experiments. ns, not significant versus NS siRNA-transfected BMDMs (Student’s *t*-test). The efficiency of the knockdown of NLRP3 and GSDMD was confirmed by WB. **e** WT or MAVS^−/−^ BMDMs were infected with VSV M or SindV for 8 or 16 h. IFNβ release in the cell supernatant was then assessed by ELISA. The data shown are means ± SD from three independent experiments. ****P < 0.0001, ***P < 0.001, versus WT BMDMs (Student’s *t*-test). **f** WT or MAVS^−/−^ BMDMs were infected with VSV M or SindV for 24 h, then cell death was assessed using the LDH release assay. The data shown are means ± SD from three independent experiments. **0.001 < *P* < 0.01, versus WT BMDMs (Student’s *t*-test). **g** Primed WT or MAVS^−/−^ BMDMs were infected with VSV M or SindV for 24 h. IL-1β release in the cell supernatant was then assessed by ELISA. The data shown are means ± SD from three independent experiments. ****P < 0.0001, **0.001 < *P* < 0.01, versus WT BMDMs (Student’s *t*-test)

Type I IFNs are not only potent antiviral cytokines preventing viral replication^34^, but they also inhibit inflammasome activation^35^. Unlike VSV, VSV M was a strong IFNβ inducer (Fig. 1f), did not replicate into BMDMs (Fig. 1h) and did not activate inflammasome (Fig. 1a–c). To further confirm that viral replication is required for NLRP3 inflammasome activation, IFNAR^−/−^ or WT BMDMs were infected with VSV M. While VSV M did not replicate in WT BMDMs, it replicated well in IFNAR^−/−^ BMDMs (Fig. 6a) since these cells are insensitive to type I IFNs. As it could replicate in IFNAR^−/−^ cells, VSV M then promoted cell death and IL-1β production (Fig. 6b, c). Likewise, SindV also replicated only in IFNAR^−/−^ BMDMs triggering, to a lesser extent, cell death and inflammasome activation (Fig. 6a–c). In IFNAR^−/−^ BMDMs, cell death induced by VSV M or SindV was not prevented by the knockdown of NLRP3 or GSDMD, suggesting that this cell death is not dependent on the inflammasome (Fig. 6d). Both VSV M and SindV did not activate inflammasome in BMDMs because their sensing leads to the production of type I IFNs preventing therefore their replication. Indeed, both viruses are sensed by the RLRs because the absence of the adaptor MAVS completely abrogated the production of IFNβ following viral infection (Fig. 6e). Hence, due to the lack of type I IFNs production in MAVS^−/−^ BMDMs, VSV M, and SindV induced-cell death allowed IL-1β release consequently of the inflammasome activation (Fig. 6f, g). Together, our observations suggest that viral replication-induced cell death is a signal allowing inflammasome activation after RNA virus infection.

## Discussion

In this study, using different RNA viruses, we observed that only replicating viruses with a cytopathogenic effect like VSV or EMCV are capable of inducing a significant inflammasome activation. In agreement with previous studies^11,13–15^, the NLRP3 inflammasome is engaged in the sensing of both viruses. Indeed, IL-1β release following VSV or EMCV infection was severely impaired in the absence of NLRP3, ASC, or caspase-1.

Pathogen recognition receptors (PRRs) sense pathogen-associated molecular patterns (PAMPs) to initiate an innate immune response^36^. Viral RNAs are the main PAMPs that are sensed by the PRRs like the RLRs (RIG-I and MDA-5) to induce the production of type I IFNs and pro-inflammatory cytokines^21^. With the exception of VSV, the different viruses triggered in BMDMs IFNβ production which was blunted in the absence of MAVS (Fig. S10), the mitochondrial adaptor downstream of RIG-I or MDA-5 required for the signaling, meaning that viral RNAs were sensed by the RLRs. The viral sensing by those helicases does not seem to be required for the inflammasome activation as SeV M or VSV M, while both were strong IFNs inducers as a consequence of RLR stimulation, did not trigger inflammasome activation after infection with BMDMs. Our results are in contradiction with a study by Poeck et al.^37^. Indeed, they have proposed a role for RLRs in NLRP3 inflammasome activation after infection with RNA viruses. Based on their results, MDA5 plays an essential but undefined role in NLRP3 inflammasome activation by EMCV. Moreover, they have proposed that following VSV infection, the virus is detected by a RIG-I/ASC/caspase-1 inflammasome that does not require NLRP3. Our results do not support this model as we have constantly observed the requirement for NLRP3 in response to VSV and EMCV, and, on the other hand, the RLRs did not seem to be required for inflammasome activation in response to these viruses. We have currently no explanations to clarify this discrepancy but our conclusions are in complete agreement with the study of Rajan et al.^11^. Furthermore, two other works have also shown the need of NLRP3 after VSV infection^11,13^. It has also been described that the DHX33 (also called DDX33) RNA helicase senses cytosolic RNA and activates the NLRP3 inflammasome^27^. However, on the other hand, the knockdown of this helicase in BMDMs did not affect inflammasome activation after VSV or EMCV infection. In agreement with another study^13^, the knockdown of DHX33 in THP-1 cells did not impair IL-1β secretion after VSV or EMCV infection either but it did after Poly(I:C) transfection, as reported^27^. A possible explanation is that an excess of nucleic acids like poly(I:C) in the cytosol after the transfection can be sensed by DHX33 to activate NLRP3 inflammasome.

**Fig. 7:**
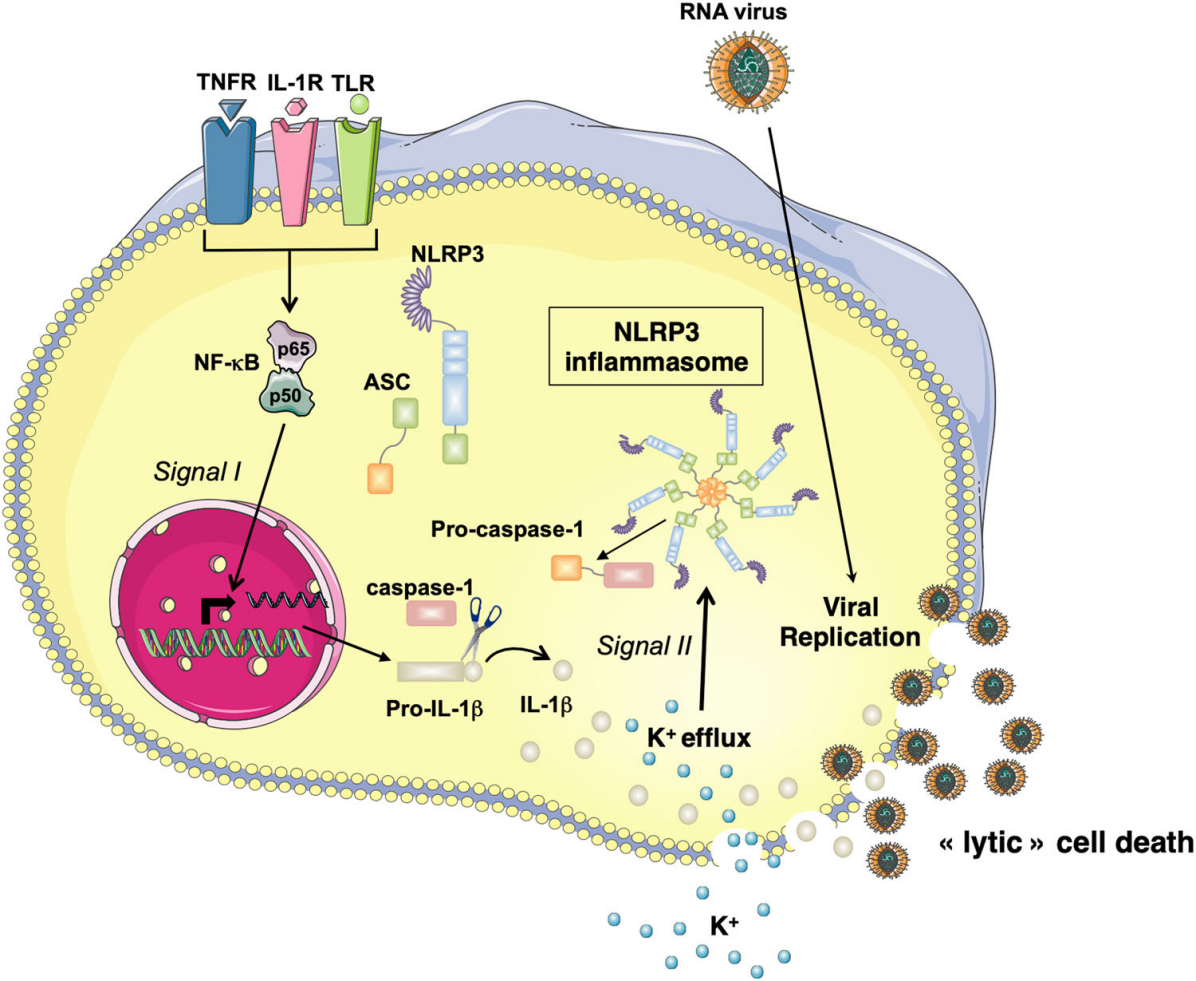
Proposed model. NF-κB activation following TLR3, TNFR, or IL-1R stimulation or following RLR stimulation (not depicted) promotes pro-IL-1β expression (signal I). In parallel, after infection, RNA virus replicates into the cell triggering a lytic form of cell death and plasma membrane rupture. This lytic cell death allows K^+^ efflux (signal II) that activates NLRP3 inflammasome. Within this inflammasome, caspase-1 is activated then cleaves pro-IL-1β into its mature form. IL-1β is released in the extracellular medium independently of the gasdermin D pores, likely as a consequence of the viral-induced lytic cell death

Based on our results, a key step for RNA viruses to induce NLRP3 inflammasome activation is their cytopathogenic effect as a consequence of their replication. Indeed, when infection with the virus did not promote cell death, either because the virus was not cytopathogenic (the case of SeV) or because it did not replicate (VSV M or SindV) after UV inactivation, the inflammasome activation was negligible. Infection with VSV or EMCV likely triggers a form of necrosis (plasma membrane rupture) as a consequence of the lytic cell death, promoting the efflux of intracellular K^+^, a necessary and sufficient upstream signaling event in NLRP3 activation^2,5^ (Fig. 7). Interestingly, similar to the infection with cytopathogenic RNA viruses, stimulation of the cGAS–STING axis in human myeloid cells induces a cell death program initiating K^+^ efflux upstream of NLRP3^38^. While both forms of cell death lead to inflammasome activation, they allow the release of IL-1β without the need of gasdermin D suggesting a plasma membrane rupture allowing first the K^+^ efflux upstream of NLRP3, then the release of mature IL-1β.

In the case of canonical NLRP3 inflammasome activation, for instance with nigericin, inflammasome activation leads to pyroptotic cell death as a consequence of gasdermin D cleavage by caspase-1. After VSV or EMCV infection, despite NLRP3 inflammasome activation, cell death was not affected in NLRP3, ASC, or caspase-1-deficient BMDMs or after treatment with a broad caspase inhibitor like zVAD-fmk, suggesting that pyroptotic or apoptotic cell death was not involved. Necroptosis is another host defense strategy to prevent viral infection^28–30^, but we did not observe MLKL phosphorylation in VSV or EMCV-infected BMDMs and the absence of MLKL did not impact the inflammasome. RIPK1 and RIPK3 are components of the necroptosome and it was recently reported that the RNA viruses promote NLRP3 inflammasome activation through a RIPK1–RIPK3–Drp1 signaling pathway^13^. In agreement with a recent study^32^, our results suggest that the RIPK1–RIPK3–Drp1 signaling pathway is not required for inflammasome activation after infection with RNA viruses. While further studies are required to explain this discrepancy, a possible explanation is that in their BMDMs deficient for RIPK1, RIPK3, or Drp1, the replication of RNA viruses was reduced, promoting less lytic cell death and thus less NLRP3 inflammasome activation^13^.

After infection, viral RNAs are sensed by the RLRs in the cytosol and through the mitochondrial adaptor MAVS, they engaged a signaling pathway leading to type I IFNs production and these cytokines combat viral infection for instance by preventing virus replication^21^. Hence, infection of BMDMs with VSV M or SindV led to IFNβ production that blocked the replication of both viruses and there was no sign of inflammasome activation. In MAVS or IFNAR^−/−^ BMDMs, VSV M or SindV replicated well because of a deficiency in IFNβ production or the absence of this cytokine receptor. Then, the viral replication triggered lytic cell death, K^+^ efflux-induced NLRP3 inflammasome activation, and ensuing the production of the highly inflammatory cytokines IL-1β and IL-18.

Hence depending on the RNA virus nature, the innate immune system deploys two strategies: on one hand, viral RNAs detection by the RLRs in the cytosol leads to type I IFNs production, on the other hand, the viruses that escape to the IFN response can replicate and trigger lytic cell death leading to IL-1β and IL-18 production through the NLRP3 inflammasome.

## Materials and methods

### Cell culture

BHK-21 and L929 cells were cultured in DMEM high glucose with 10% FBS. For the mouse BMDMs, the bone marrow was extracted by flushing from the femurs and tibia of mice. Bone marrow was incubated in DMEM with 30% of L929-conditioned medium, 10% FBS, 1% penicillin and streptomycin, and 2 mM l-glutamine for 7 days before using the differentiated macrophages for experiments. Bone marrow from NLRP3^−/−^, RIPK3^−/−^, or MLKL^−/−^ mice were obtained from Dr. James Vince (WEHI Institute, Melbourne, Australia), IFNAR^−/−^ from Dr. Damien Vitour (LabEx IBEID, Maisons-Alfort, France), CASP-1/CASP-11^−/−^ from Dr. Richard Flavell (Department of Immunobiology, Yale University School of Medicine, USA), MAVS^−/−^ from Dr. Marie-Cécile Michallet (Centre International de Recherche en Infectiologie, INSERM U1111-CNRS UMR5308, Lyon, France), ASC^−/−^ from Dr. Ronan Le Goffic (Unité de Virologie et Immunologie Moléculaires, UR 892 INRA, Jouy-en-Josas, France), GSDMD^−/−^ from Dr. Petr Broz (Faculty of Biology and Medicine, Department of Biochemistry, Université de Lausanne, Switzerland), RIG-I^−/−^ from Dr. Winfried Barchet (Translational Immunology Institute of Clinical Chemistry and Clinical Pharmacology, University of Bonn, Germany), and MDA5^−/−^ from the Jackson Laboratory. All the mice were C57Bl/6 background except RIG-I mice that were CD1 (ICR) background.

### Viral Infections and cell treatments

The SeV M and the VSV wild type and mutant (VSV-M) were kindly provided by Dr. Dominique Garcin (Department of Microbiology and Molecular Medicine, Faculty of Medicine, University of Geneva, Geneva, Switzerland). The SindV was kindly provided by Dr. Marco Vignuzzi (Institut Pasteur, Paris, France). Briefly, BMDMs cells were washed once with 1⨯ PBS and incubate with VSV, VSV-M, EMCV, SeV, SeV-M, and SindV at a MOI of 5 for 2 h at 37 C in 5% CO_2_. Then, the medium with a non-adsorbed viruses was removed by washing, the cells were washed with serum-free medium and cultured in DMEM supplemented with 5% FBS, at 37 °C in 5% CO_2_. After different time post-infection, conditioned media were collected for virus titration, necrosis, and cytokine quantification and some western blot analyses. Whole cell extracts were used for western blot analyses. As a control of virus replication, viruses were UV inactivated by irradiation for 5 min in a Stratagene 2400 UV cross-linker.

In some experiments, BMDMs were treated with zVAD-fmk (10 μM) (Invivogen), Nigericin (10 μM) (Invivogen), ATP (5 mM) (Invivogen), and glyburide (25 μg/ml) (Invivogen). BMDMs were primed with ultrapure LPS (100 ng/ml) (Invivogen).

### Virus quantification

The plaque assay—SindV, EMCV, and VSV infectious particles released from infected cells were quantified by determining virus titers in the culture medium by the plaque assay in BHK-21 cells, as previously described^39^. Plaques were visualized by staining the monolayer with 1 ml 1% crystal violet in 20% ethanol. SeV replication was assessed by flow cytometry as the virus contains a GFP-tag.

### Cell death assay

Cell death was assessed through a lactate dehydrogenase (LDH) released from infected macrophages. The LDH release was determined by the Promega CytoTox 96 assay kit (Promega) according to the manufacturer’s instructions.

### Enzyme-linked immunosorbent assays (ELISA)—cytokines quantifications

The concentrations of cytokines in the conditioned medium of BMDM cultures were determined by ELISA. IL-1β (Thermo Fisher Scientific), IL-18 (MBL International), IFN-β (PBL Assay Science) concentration was quantified using the standard ELISA development kit according to the manufacturer’s protocol.

### Protein extraction and immunoblots

Cells were lysed in lysis buffer (50 mM Tris–HCl pH 7.4, 150 mM NaCl, 1% Triton X-100, 2 mM EDTA, 2 mM sodium pyrophosphate, 25 mM β-glycerophosphate, 1 mM sodium orthovanadate) supplemented with protease inhibitor cocktail (Thermo Fisher Scientific), and the debris were removed by centrifugation at 10,000 × *g* and 4 °C. Protein concentration was determined with a micro-BCA kit (Thermo Fisher Scientific). Samples were then boiled in SDS sample buffer (Novex) containing 10% β-mercaptoethanol (Sigma) and resolved by SDS–polyacrylamide gel electrophoresis. Immunoblot analysis was performed with specific antibodies and the antigen**–**antibody complexes were visualized by chemiluminescence (Immobilon Western, Merck Millipore).

### Antibodies

The primary antibodies used for immunoblotting were mouse IgG1 anti-caspase-1 (p20) (Adipogen, #AG-20B-0042, 1/2000 dilution), mouse IgG2b anti-NLRP3 (Adipogen, #AG-20B-0014, 1/1000), goat anti-mouse IL-1β (R&D systems, #AF-401-NA, 1/1000), rabbit anti-GAPDH (Sigma-Aldrich, #G9545, 1/20,000), rabbit anti-IRF3 (Cell Signaling, #4302, 1/2000), rabbit anti-phospho-IRF3 (Ser396) (Cell Signaling, #4947, 1/2000), rabbit anti-MAVS (rodent specific) (Cell Signaling, # 4983, 1/1000), rabbit anti-phospho-STAT1 (Tyr701) (Cell Signaling, #9171, 1/1000), rabbit anti-STAT1 (D1K9Y) (Cell Signaling, #14994, 1/3000), rabbit anti-caspase-3 (Cell Signaling, #9662, 1/1000), mouse IgG1 anti-DDX33 (B-4) (Santa Cruz, #sc-390573, 1/1000), rabbit anti-MLKL (phospho S345) (Abcam, #ab196436, 1/1000), rabbit anti-MLKL (D6W1K) (Cell Signaling, #37705, 1/2000), rabbit anti-phospho-DRP1 (Ser616) (D9A1) (Cell Signaling, #4494, 1/1000), mouse IgG1 anti-DRP1 (BD Biosciences, #611113, 1/2000), rabbit anti-RIPK1 (D94C12) (Cell Signaling, #3493, 1/2000), and guinea pig anti-mouse gasdermin D (Adipogen, #AG-25B-0036, 1/1000).

### Transfection with siRNA

BMDMs were transfected with small interfering RNAs. Briefly, cells were plated in 48-well plates (at a density of 5 × 10^5^ cells per well) and then were transfected with 50 nM siRNA through the use of INTERFERin (Polyplus) according to the manufacturer’s guidelines. Control nonspecific siRNAs and the specific siRNAs were purchased from Sigma-Aldrich. The siRNAs used were: Drp1 a (5′GGAAUAAUUGGAGUAGUUAdTdT3′), Drp1 b (CUGUCAAUUUGCUAGAUGUdTdT), DDX33 a (GCAAGAAUAUGCUGCUAGUdTdT), DDX33 b (CCCAAAUGUGCUCACCUUUdTdT), RIPK1 a (CACAAUCCUUUCUUACACAdTdT), RIPK1 b (GGAAGAUAUUGUGAGCGGAdTdT), NLRP3 a (GAUCAACCUCUCUACCAGAdTdT), NLRP3 b (GUGUUGUCAGGAUCUCGCAdTdT), GSDMD a (GAUUGAUGAGGAGGAAUUAdTdT), GSDMD b (CUGCUUAUUGGCUCUAAAUdTdT).

### ASC speck immunofluorescence

BMDMs were plated at 5 × 10^5^ cells per well in 24-well plates on sterile glass coverslips. Cells were fixed by incubation in 4% paraformaldehyde in phosphate buffered saline (PBS) for 10 min, and then permeabilized by incubation with 0.15% Triton X-100 in PBS for 15 min. Nonspecific-binding sites were blocked by incubating cells in a solution of 2% BSA in PBS for 1 h. The cells were then incubated overnight at 4 °C with the rabbit mAb anti-ASC mouse specific (D2W8U) (Cell Signaling, #67824, 1/400 dilution). They were washed three times, for 5 min each, in PBS and were then incubated for 1 h with the specific Alexa Fluor-conjugated secondary antibodies (Invitrogen). Nuclei were stained with DAPI (Sigma) and cells were again washed three times with PBS. Images were acquired with a Leica SP5 confocal microscope (Leica Microsystems) equipped with a ×63 oil immersion fluorescence objective.

### ASC oligomerization

BMDMs were seeded in 24-well plates at 1.0 × 10^6^ cells/well. After appropriate treatments, cells were lysed with cold PBS containing 0.5% Triton X-100, and the cell lysates were centrifuged at 6000 × *g* for 15 min at 4 °C. The pellets were washed twice with PBS and then resuspended in 200 μl PBS. Freshly prepared disuccinimidyl suberate (2 mM) was added to the resuspended pellets and the suspension was incubated at room temperature for 30 min with rotation. The cross-linked pellets were collected by centrifugation at 6000 × *g* for 15 min at 4 °C and redissolved in 25 μl of 1× SDS–PAGE sample loading buffer. Samples were boiled for 5 min and subjected to western blot analysis.

### Generation of THP-1 cells expressing shRNA

shRNAs targeting mRNA of DHX33 were from Sigma.

shDHX33 (1): CATTTCCTTTAGAACCCAAAT; shDHX33 (2): GTTGACACGGGCATGGTTAAA.

A PLKO.1 vector encoding shRNA for a scrambled (Sigma) or DHX33 was transfected into HEK293T cells together with psPAX2, a packaging plasmid, and pMD2.G, an envelope plasmid, for producing viral particles with Lipofectamine 2000 (Life Technologies). Culture supernatants were harvested 48 h after transfection, filtered through a 0.45 nm filter and concentrated with PEG-IT (Cell Signaling). THP-1 cells were infected with collected supernatants containing lentiviral particles in the presence of 4 μg/ml polybrene (Sigma). After 48 h of culture, lentiviral-infected cells were selected by 5 μg/ml puromycin (Invivogen).

### Stimulation of THP-1 macrophages

THP-1 cells were differentiated into macrophages with 60 nM phorbol 12-myristate 13-acetate (PMA; Sigma) for 16 h, and cells were cultured for an additional 48 h without PMA. Differentiated cells were stimulated for 15 h with 1.0 μg/ml poly(I:C) (Invivogen) plus Lipofectamine 2000 or infected with VSV or EMCV.

#### Statistical analysis

We carried out *t* tests with Tukey’s post hoc analysis to assess the statistical significance of differences (Prism GraphPad Software), and the *P* values obtained are indicated in the figure legends. Differences were considered to be significant if *P* < 0.05. ****P < 0.0001, ***P < 0.001, **0.001 < *P* < 0.01, *0.01 < *P* < 0.05. ns, not significant. The data shown in each histogram are the means ± SD from three independent experiments.

## Supplementary information

Figure S1

Figure S2

Figure S3

Figure S4

Figure S5

Figure S6

Figure S7

Figure S8

Figure S9

Figure S10

Supplementary figure legends
