# Supplementary figures and images for: RNA viruses promote activation of the NLRP3 inflammasome through cytopathogenic effect-induced potassium efflux
Source: Cell Death Dis. 2019 Apr 25;10(5):346. doi: 10.1038/s41419-019-1579-0 (PMC6483999; doi:10.1038/s41419-019-1579-0)

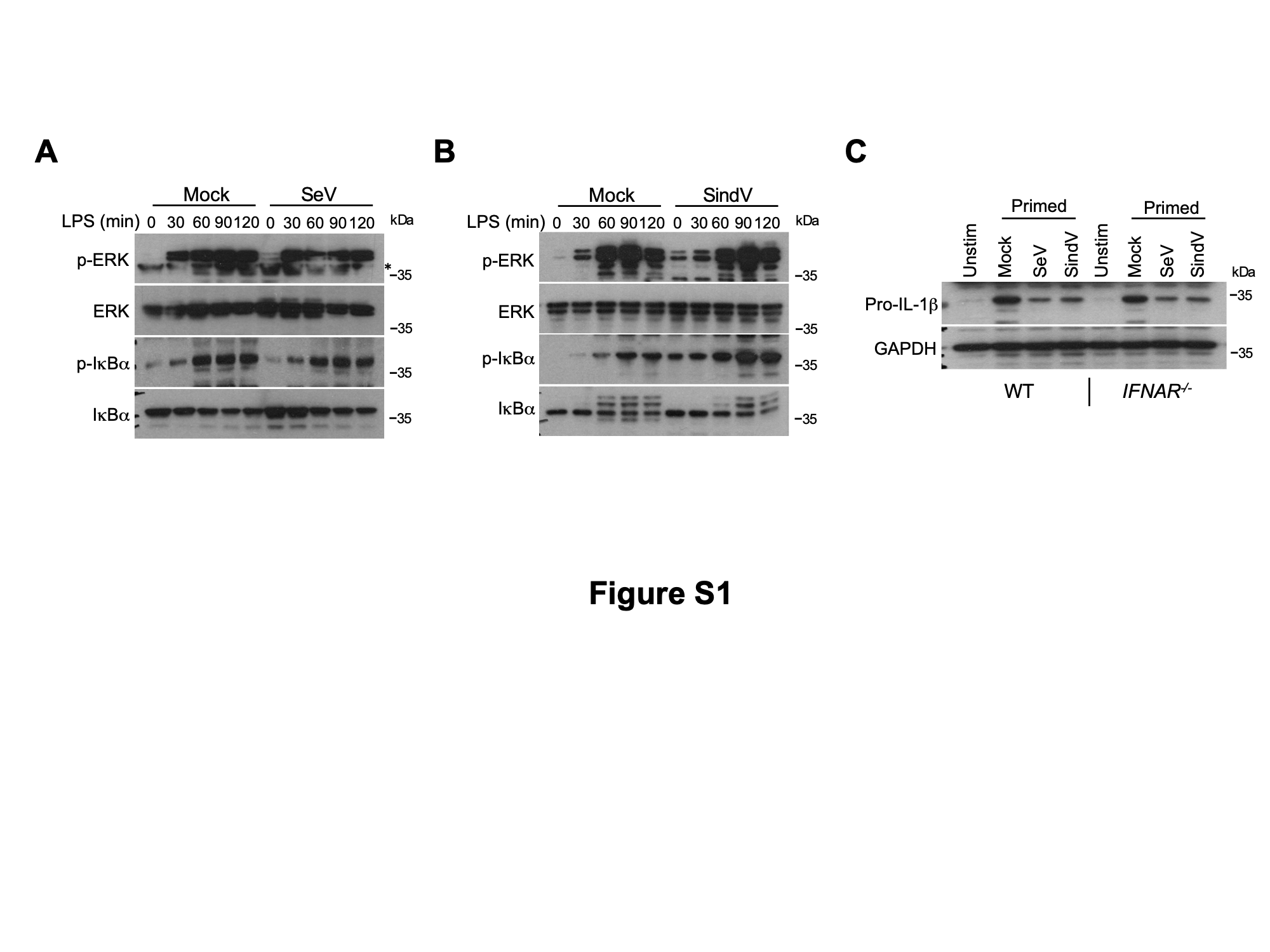

Supplement: Supplementary file 1 — Figure S1 [file 41419_2019_1579_MOESM1_ESM.tif]

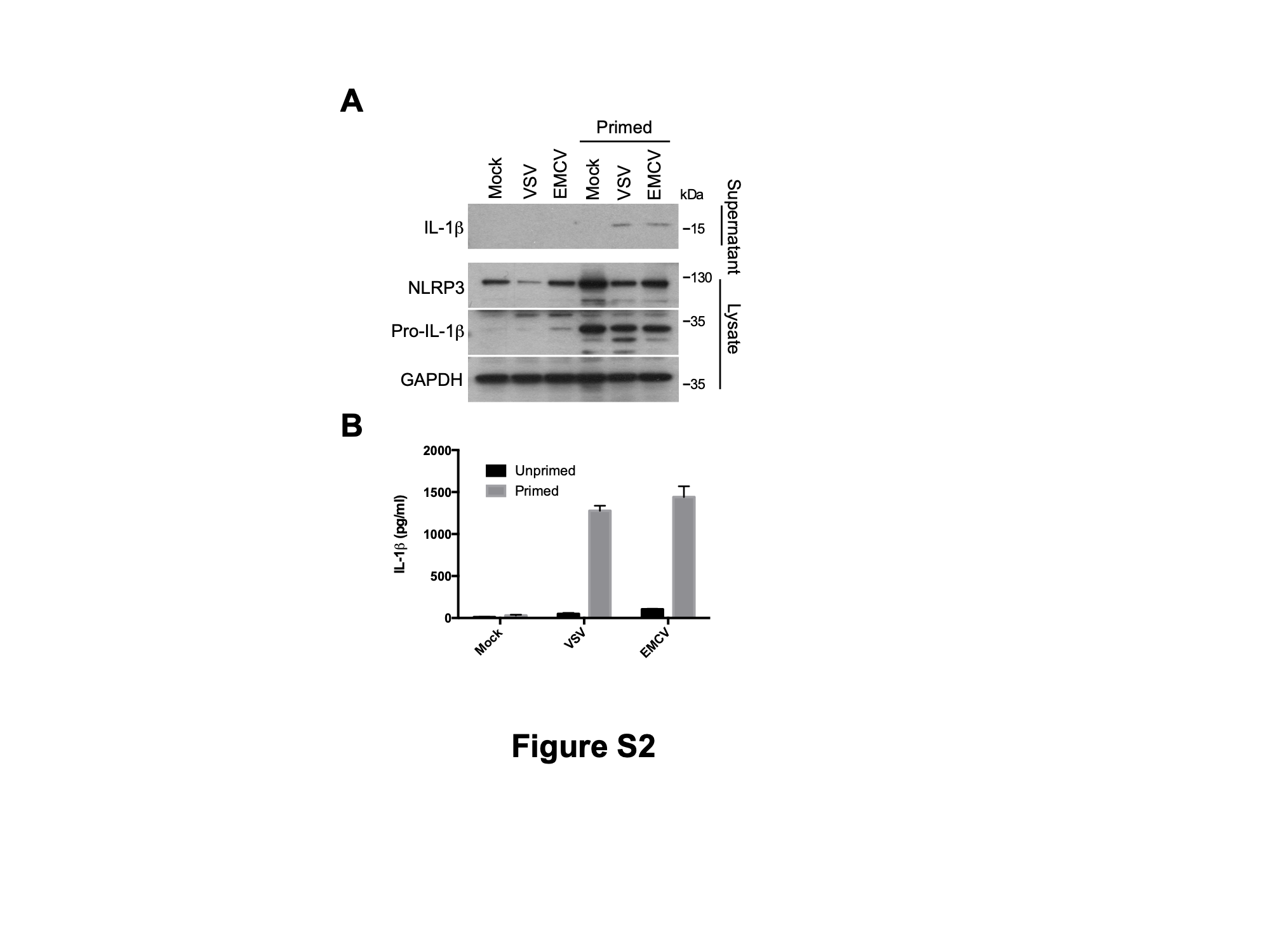

Supplement: Supplementary file 2 — Figure S2 [file 41419_2019_1579_MOESM2_ESM.tif]

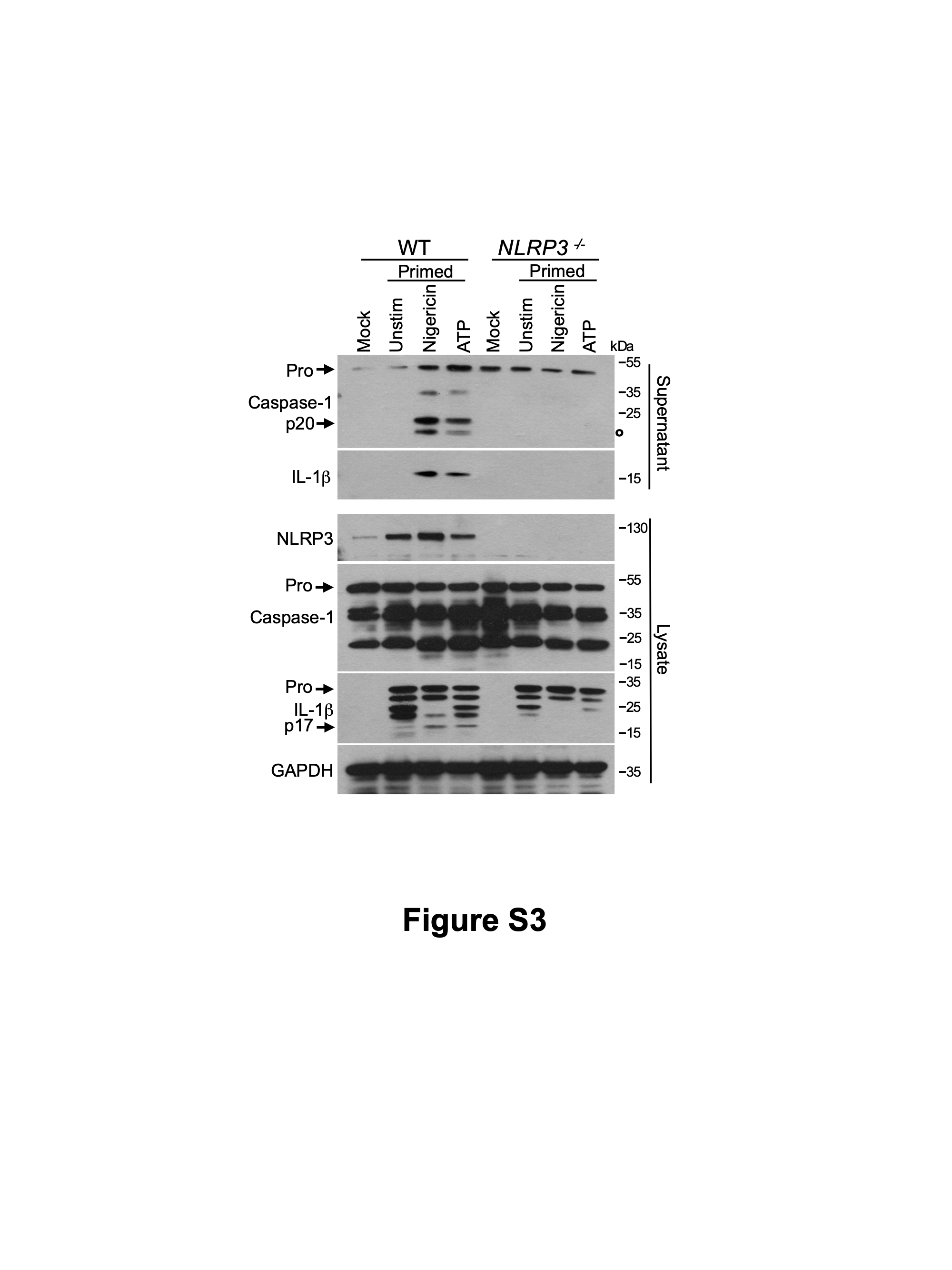

Supplement: Supplementary file 3 — Figure S3 [file 41419_2019_1579_MOESM3_ESM.tif]

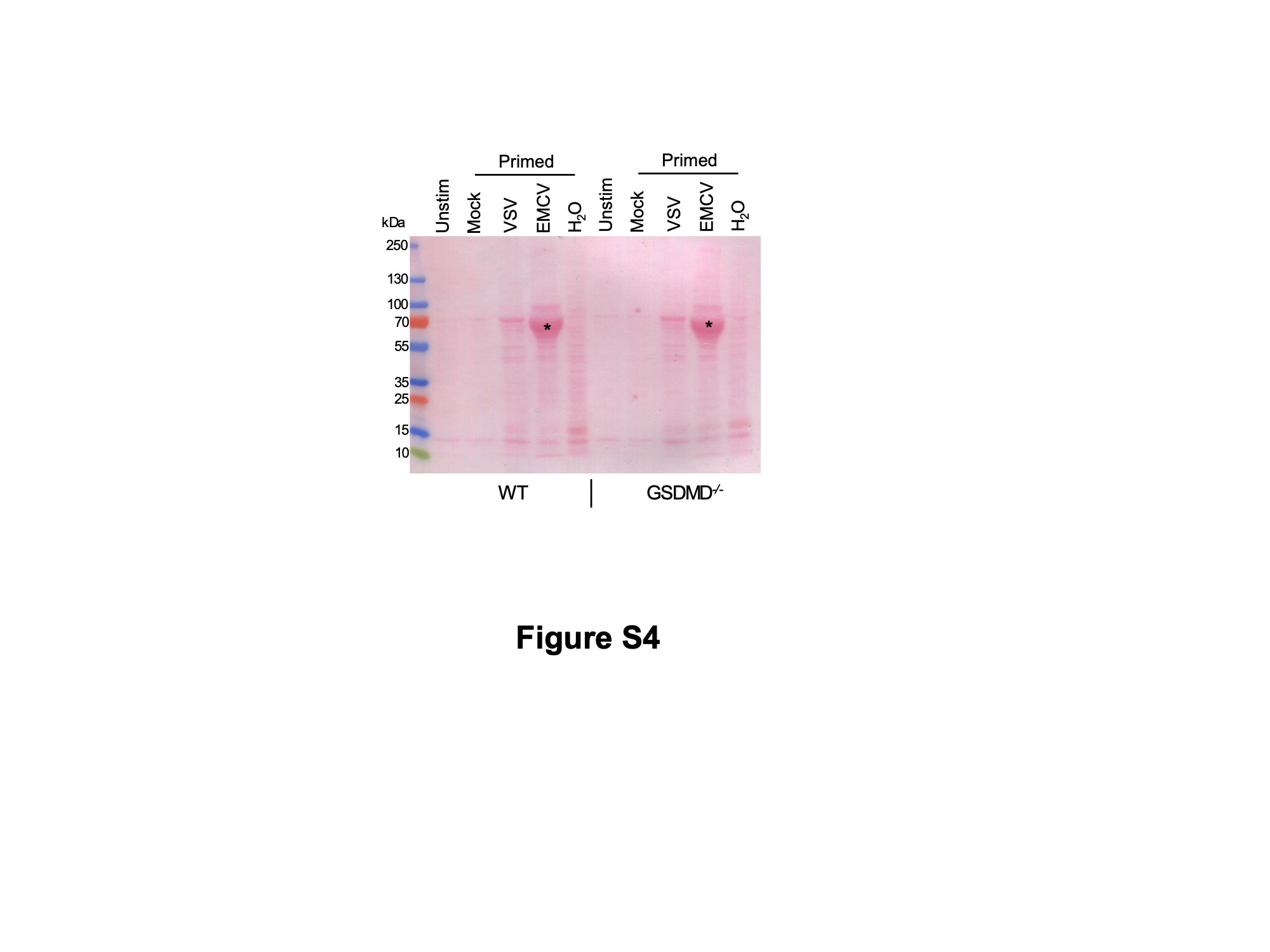

Supplement: Supplementary file 4 — Figure S4 [file 41419_2019_1579_MOESM4_ESM.tif]

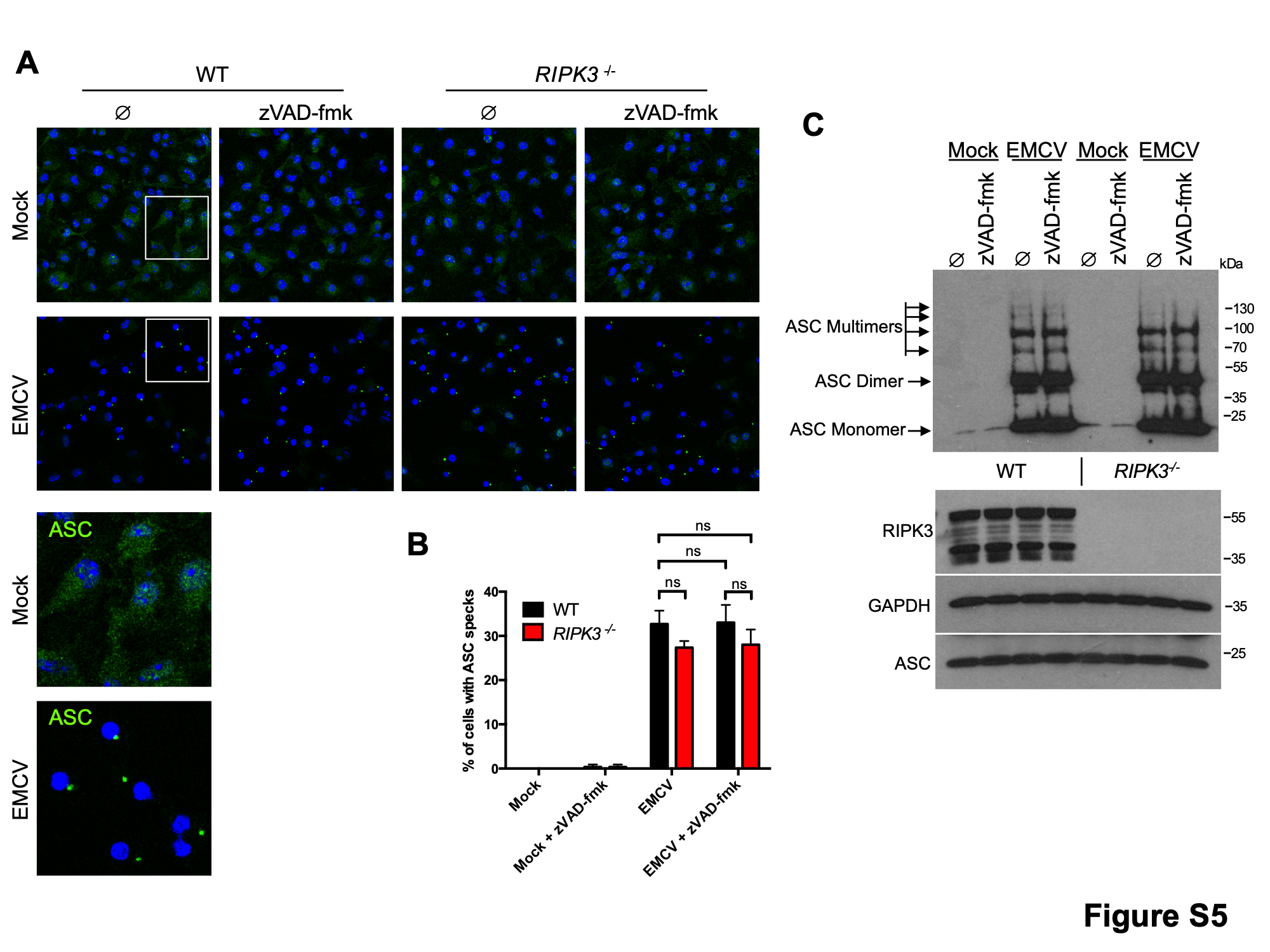

Supplement: Supplementary file 5 — Figure S5 [file 41419_2019_1579_MOESM5_ESM.tif]

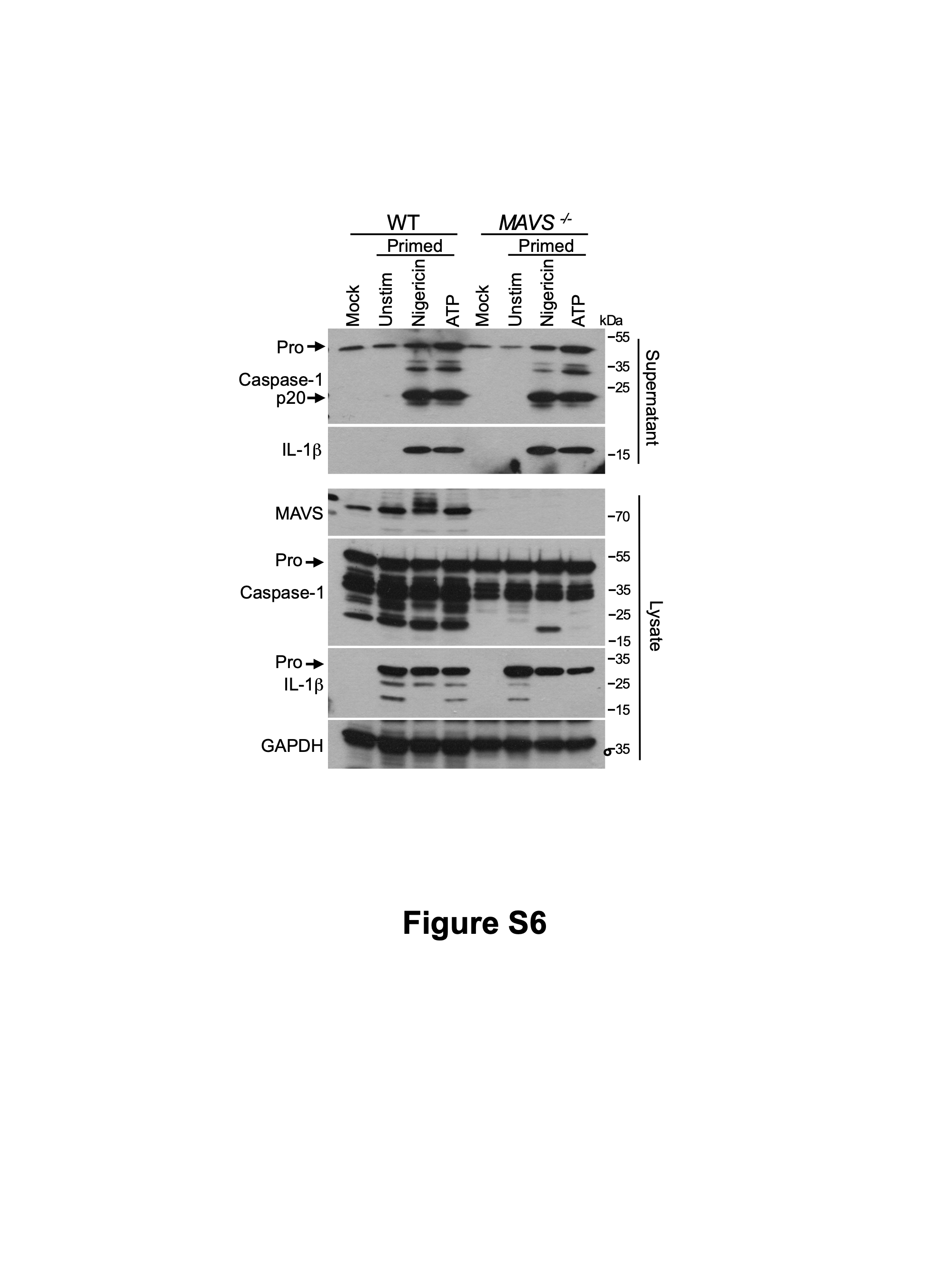

Supplement: Supplementary file 6 — Figure S6 [file 41419_2019_1579_MOESM6_ESM.tif]

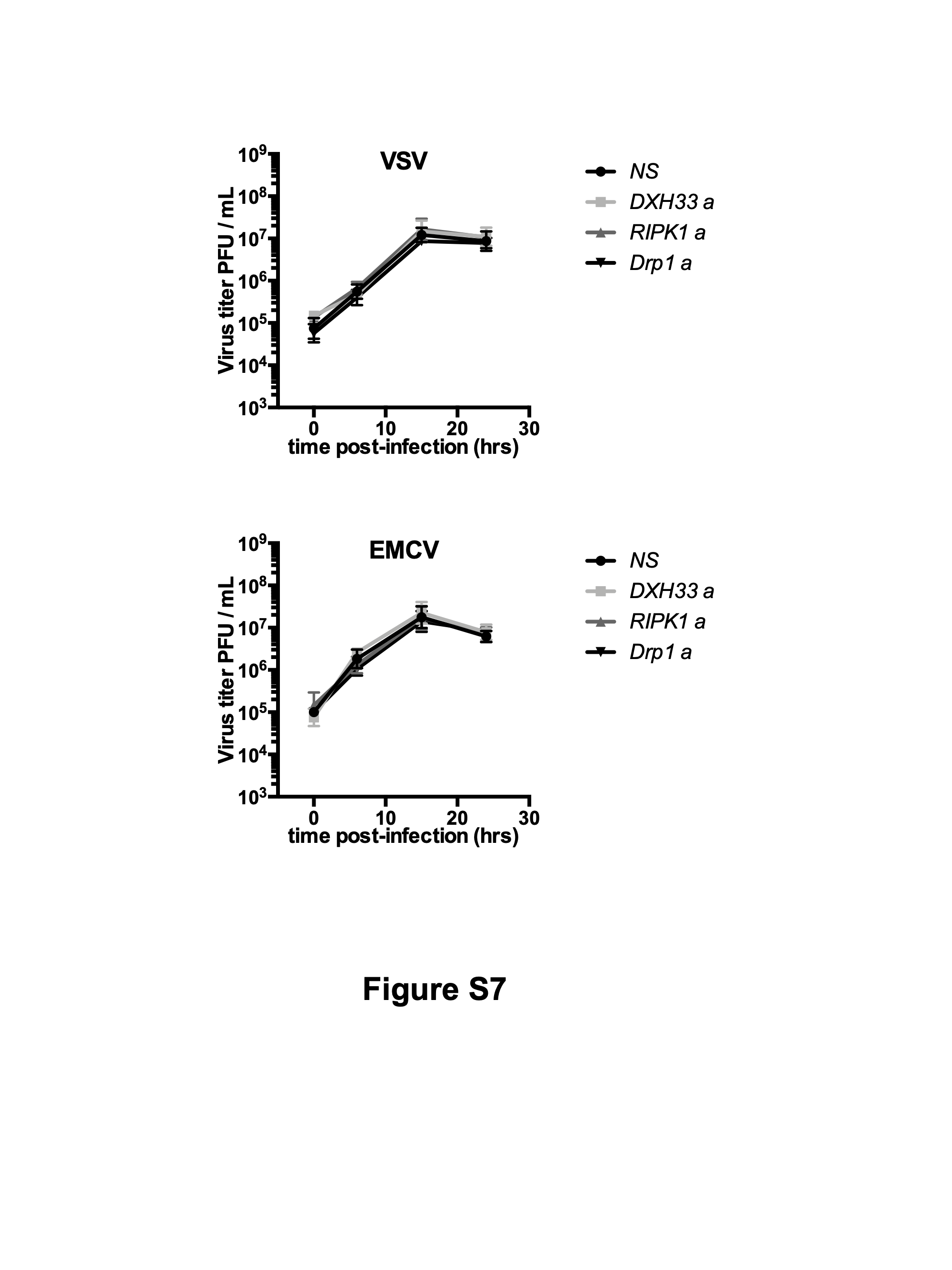

Supplement: Supplementary file 7 — Figure S7 [file 41419_2019_1579_MOESM7_ESM.tif]

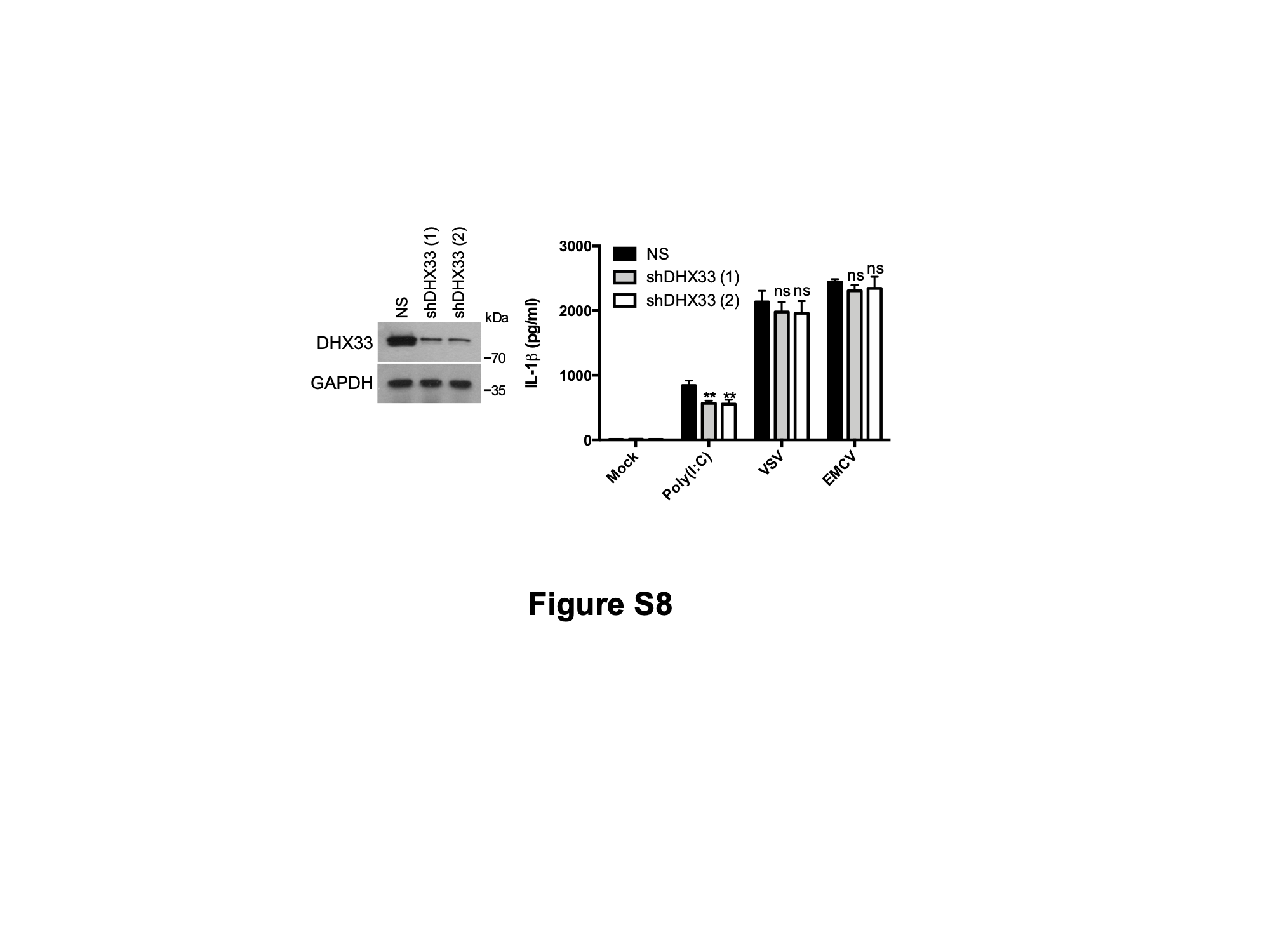

Supplement: Supplementary file 8 — Figure S8 [file 41419_2019_1579_MOESM8_ESM.tif]

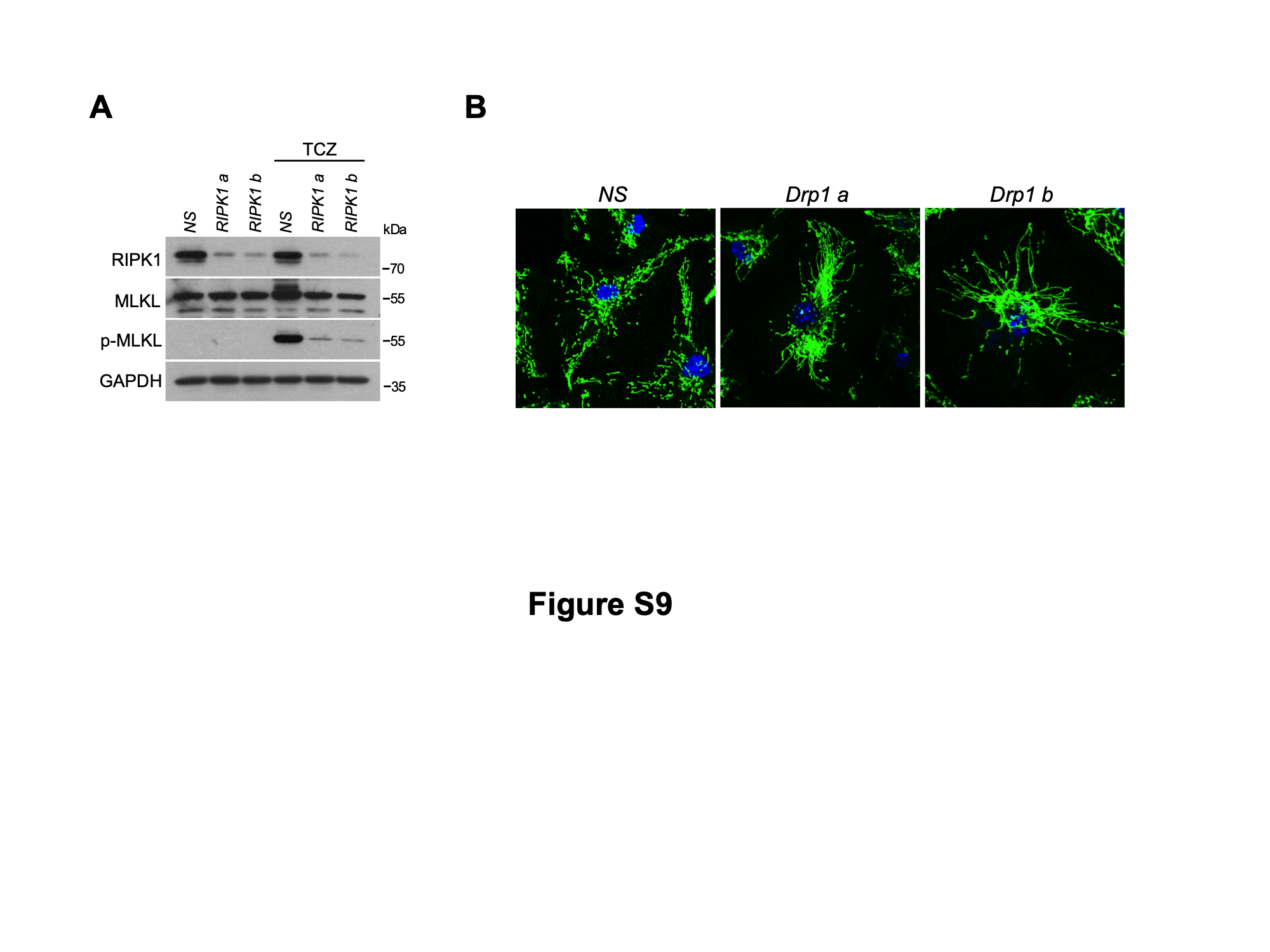

Supplement: Supplementary file 9 — Figure S9 [file 41419_2019_1579_MOESM9_ESM.tif]

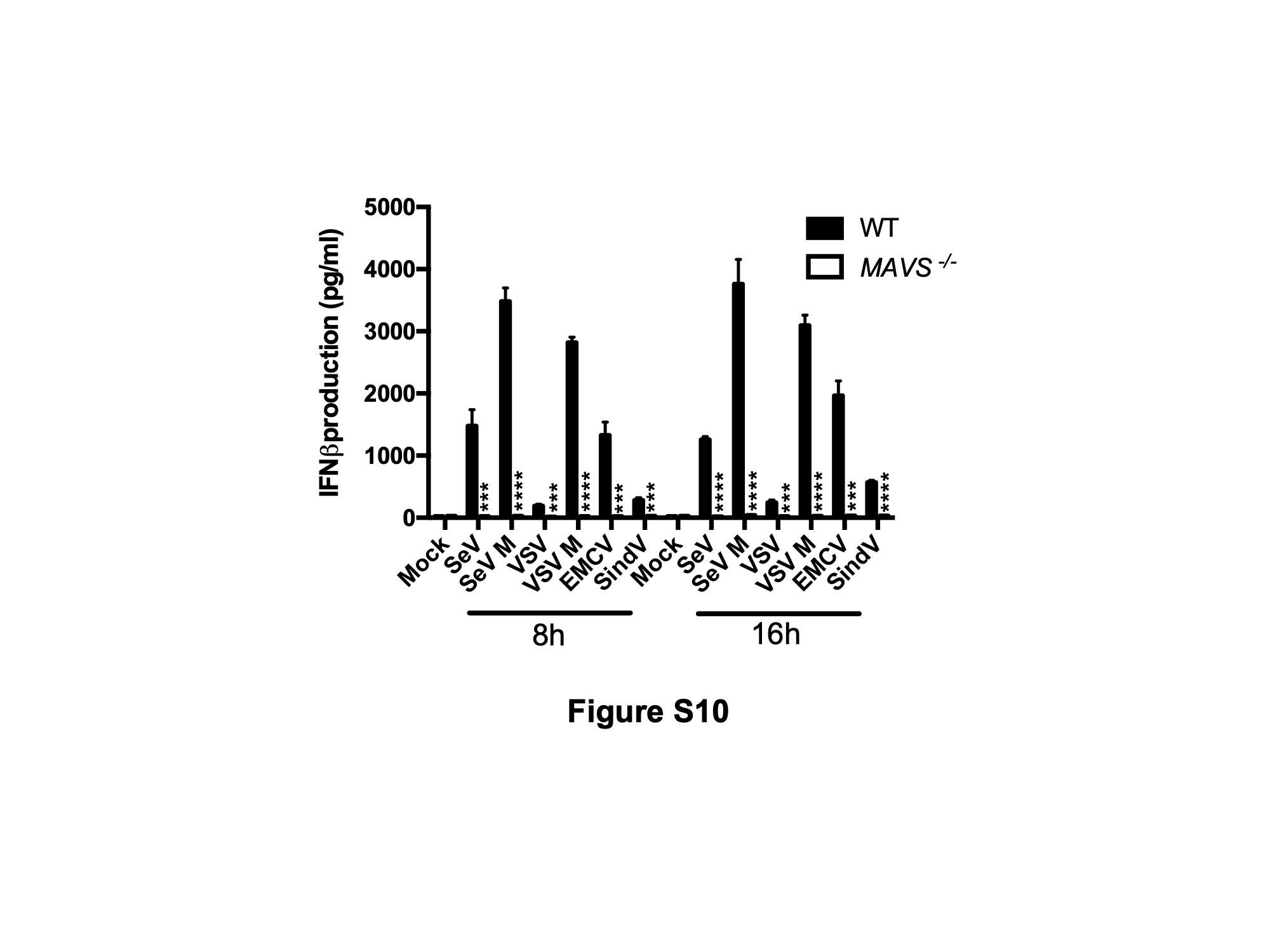

Supplement: Supplementary file 10 — Figure S10 [file 41419_2019_1579_MOESM10_ESM.tif]
