# Supplementary material for: RNA viruses promote activation of the NLRP3 inflammasome through cytopathogenic effect-induced potassium efflux
Source: Cell Death Dis. 2019 Apr 25;10(5):346. doi: 10.1038/s41419-019-1579-0 (PMC6483999; doi:10.1038/s41419-019-1579-0)
Supplement: Supplementary file 11 — Supplementary figure legends [file 41419_2019_1579_MOESM11_ESM.docx]

**Figure S1: Sendai and Sindbis viruses do not inhibit TLR4 signaling**

BMDMs were infected with SeV (A) or SindV (B) for 16 hrs. Cells were then stimulated with LPS (100 ng/ml) for the indicated times and cell lysates were analyzed by WB for the indicated proteins. (C) Primed (LPS, 100 ng/ml for 3 hrs) WT or IFNAR^-/-^ BMDMs were infected with SeV or SindV for 16 hrs. Then, cell lysates were analyzed by WB for the indicated proteins.

**Figure S2: Priming is required for VSV and EMCV to activate NLRP3 inflammasome**

(A) Unprimed or primed BMDMs were infected with VSV or EMCV for 15 hrs. Then, cell supernatants and cell lysates were analyzed by WB for the indicated proteins. (B) Unprimed or primed BMDMs were infected with VSV or EMCV. 15 hrs later, IL-1β release in the cell supernatant was assessed by ELISA.

**Figure S3: Nigericin and ATP trigger NLRP3 inflammasome activation**

Primed WT or NLRP3^-/-^ BMDMs were treated with nigericin (10 uM) or ATP (5 mM) for 45 min. Then, cell supernatants and cell lysates were analyzed by WB for the indicated proteins.

**Figure S4: VSV or EMCV infection promotes plasma membrane rupture leading to the release of cytosolic proteins**

Primed (LPS, 100 ng/ml for 3 hrs) WT or GSDMD^-/-^ BMDMs were infected with VSV or EMCV for 15 hrs. Then, cell supernatants were analyzed after ponceau red staining. As a positive control for necrosis, BMDMs were incubated 1 hr in water. * likely indicates an overproduced EMCV protein.

**Figure S5: Inhibition of apoptosis and/or necroptosis does not prevent NLRP3 inflammasome activation following EMCV infection**

(A) Primed WT or RIPK3^-/-^ BMDMs were infected with EMCV for 8 hrs in the presence or the absence of the broad caspase inhibitor zVAD-fmk. ASC specks formation was then assessed by immunofluorescence. The percentage of cells with ASC specks is indicated in (B). The data shown are means ± SD from three independent experiments. ns, not significant (Student’s t-test). (C) Same conditions as in (A) but ASC oligomerization was assessed by WB after cross-linking.

**Figure S6: MAVS is not involved in canonical NLRP3 inflammasome**

Primed WT or MAVS^-/-^ BMDMs were treated with nigericin (10 uM) or ATP (5 mM) for 45 min. Then, cell supernatants and cell lysates were analyzed by WB for the indicated proteins.

**Figure S7: The knock down of DHX33, RIPK1 or Drp1 does not impact viral replication**

VSV or EMCV replication was assessed in NS, DHX33, RIPK1 or Drp1 siRNA-transfected BMDMs.

**Figure S8: The knock down of DHX33 inhibits IL-1β release after poly(I:C) transfection but not after VSV or EMCV infection in THP-1 cells**

THP-1 cells stably expressing non specific (NS) shRNA or shRNA raised against DHX33 were either transfected by poly(I:C) (1μg/ml) or infected with VSV or EMCV for 15 hrs. IL-1β release was then assessed by ELISA. The data shown are means ± SD from three independent experiments. **0.001 <P < 0.01, ns, not significant versus NS shRNA expressing THP-1 cells (Student’s t-test). The efficiency of the knock down of DHX33 was confirmed by WB.

**Figure S9: Validation of the knock down of RIPK1 or Drp1 with siRNAs in BMDMs.**

(A) BMDMs were transfected with siRNAs raised against RIPK1 or non specific siRNA (NS). 3 days later, BMDMs were stimulated with TCZ for 2 hrs. Cell lysates were then analyzed by WB for the indicated proteins. (B) BMDMs were transfected with siRNAs raised against Drp1 or non specific siRNA (NS). 3 days later, mitochondrial morphology was assessed by immunofluorescence using anti-Tom20 staining.

**Figure S10: MAVS is required for IFNβ production after RNA virus infection**

WT or MAVS^-/-^ BMDMs were infected with different RNA viruses for 8 or 16 hrs. Then, IFNβ production was assessed by ELISA. The data shown are means ± SD from three independent experiments. ****P < 0.0001, ***P < 0.001, versus WT BMDMs (Student’s t-test).
